# Towards the One Exposome: harnessing artificial intelligence and digital health technologies across One Health toxicology, new approach methodologies, and environmental public health

**DOI:** 10.3389/fpubh.2026.1921273

**Published:** 2026-10-06

**Authors:** Sandra Coecke, Thomas Hartung

**Affiliations:** 1 European Commission, Joint Research Centre (JRC), Ispra, Italy; 2 Center for Alternatives to Animal Testing, Environmental Health and Engineering Department, Bloomberg School of Public Health, Johns Hopkins University, Baltimore, MD, United States; 3 CAAT-Europe, University of Konstanz, Konstanz, Germany

**Keywords:** artificial intelligence, digital environmental health, Exposome, microphysiological systems, new approach methodologies, One Exposome, One Health toxicology, public health governance

## Abstract

The One Exposome framework integrates exposomics, One Health toxicology, New Approach Methodologies (NAMs) and artificial intelligence to advance environmental health from reactive risk assessment towards predictive, preventive, and participatory science. The paper positions One Health as the ethical and conceptual rationale for recognizing the interdependence of human, animal, and environmental health, the Exposome as the framework for measuring cumulative and life-course exposures, and NAMs together with digital and AI-enabled tools as the means to generate, integrate, and interpret mechanistic and adverse outcome pathway (AOP)-based evidence across biological scales. In this review, environmental health challenges such as antimicrobial resistance, endocrine disruption, and other complex multi-stressor exposures cannot be adequately addressed through compartmentalized, single-substance, or purely retrospective approaches. The One Exposome paradigm instead proposes a systems-based model that links exposure data, mechanistic toxicology, and public health intelligence across humans, animals, and ecosystems. It draws on human-relevant NAMs, including microphysiological systems, together with AI and multi-omics approaches, to support cross-species interpretation, earlier hazard identification, and more preventive intervention strategies. Within this framework, sentinel surveillance, integrated monitoring, and interoperable data systems are central for detecting shared risks across environmental, veterinary, food, and human health domains. The approach is also presented as requiring governance reform so that agricultural practice, environmental management, and clinical stewardship are aligned within a common preventive and planetary health perspective. Overall, the paper frames the One Exposome as a transdisciplinary architecture for environmental public health that combines measurable environments, mechanistic understanding, and computational integration. By connecting cumulative exposures to biological responses and population-level consequences, it offers a foundation for more anticipatory, equitable, and scientifically integrated environmental health decision-making.

## 1 Introduction

### 1.1 The Exposome challenge in environmental and public health

Within a decade of diclofenac’s introduction to South Asian veterinary practice, >95% of Gyps vultures had disappeared owing to renal failure after feeding on contaminated cattle carcasses (1–4). The ecological vacuum increased carcass persistence, fueling feral dog and rat populations and precipitating an estimated ≈ 100,000 excess human deaths per year from rabies and secondary infections (5, 6).^1^

What began as a pharmaceutical innovation intended to alleviate animal suffering thus cascaded into one of the most severe ecological and public health crises of modern times. The veterinary use of diclofenac, therapeutically safe for mammals but acutely nephrotoxic to vultures (Figure 1), triggered the collapse of a keystone scavenger population that had long served as India’s natural sanitation system. With carcasses remaining unconsumed for days instead of minutes, nutrient cycling, microbial decomposition dynamics, and pathogen ecology were fundamentally altered. The subsequent population explosion of opportunistic scavengers created a self-perpetuating disease reservoir that overwhelmed public health systems in rural India. The resulting rise in zoonotic transmission, especially of rabies, transformed a veterinary drug’s collateral effects into a major human mortality burden, producing measurable economic losses estimated at nearly 70 billion US$ annually (6).

**Figure 1:**
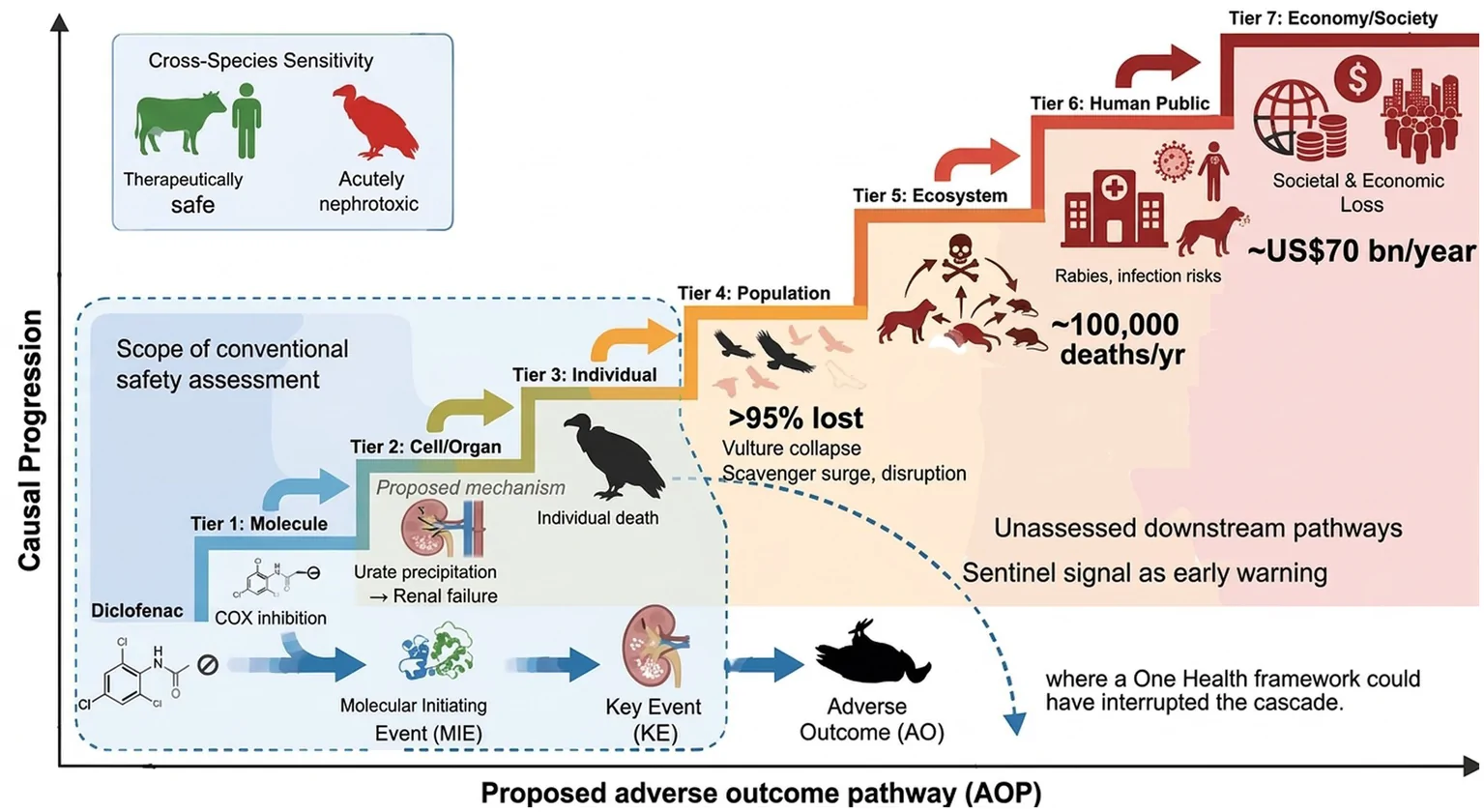
Diclofenac and the failure of compartmentalized safety assessment: An adverse outcome pathway across scales. A drug validated as safe in mammals (inset, green) is acutely nephrotoxic to *Gyps vul*tures (red). The horizontal axis shows the proposed molecular pathway (prostaglandin inhibition → urate precipitation → renal failure); the vertical axis traces consequences across levels of biological organization, from molecule to ecosystem to human public health and economy. Conventional regulatory assessment (dashed boundary) terminated at the mammalian/livestock compartment, leaving downstream scavenger, ecological, and human-health pathways unassessed. A sentinel-surveillance signal (vulture die-off as biomarker) marks where a One Health framework could have interrupted the cascade.

This chain of events linking pharmaceutical use, wildlife collapse, environmental perturbation, and human disease, represents a textbook illustration of the One Health principle: that the wellbeing of humans, animals, and ecosystems constitutes a single, interdependent continuum (7, 8). The One Health approach recognizes the intricate connections between human, animal, and environmental health, and has been widely adopted globally, with numerous organizations and governments recognizing its importance in addressing and especially governing the complex relationships between human, animal, and environmental health (9–12). This is reminiscent of the quote “The road to hell is paved with good intentions.” Often – though uncertainly – attributed to St. Bernard of Clairvaux (1090–1153).

The vulture–diclofenac crisis did not occur in isolation but reflects a broader pattern whereby chemical and biological agents traverse sectoral boundaries. The methylmercury poisoning in Minamata, Japan, where cats manifested neurological deficits before human cases emerged (13), and the rise of antimicrobial resistance (AMR) driven by aquaculture and livestock antibiotic use (14–16) similarly demonstrate how anthropogenic substances can re-enter human systems through complex ecological feedback loops. Each of these cases highlights the shared vulnerabilities created when chemicals are evaluated or regulated solely within disciplinary silos.

From a toxicological perspective, the vulture collapse exposes the fundamental limitations of reductionist safety assessment paradigms. Conventional toxicology, built upon species-specific dose/response testing and narrow hazard endpoints, rarely captures the indirect, multispecies, and ecological consequences of chemical exposure. In the case of diclofenac, existing regulatory frameworks evaluated its safety for humans and livestock but failed to account for downstream exposure pathways, such as scavenging wildlife, that determine the compound’s ultimate ecological fate. This oversight reflects a systemic blind spot: the assumption that chemical risks can be adequately managed within isolated compartments of use, neglecting their integrated environmental life cycles.

Similarly, the COVID-19 pandemic further highlighted the urgency of One Health approaches to zoonotic disease, with global mortality reaching millions and exposing gaps in cross-species surveillance (17).

### 1.2 Why the Human Exposome is no longer sufficient

The Human Exposome Project (HEP), though focused primarily on the exposure side of human disease and largely neglecting ecotoxicology, represents a natural evolution of the One Health vision. Whereas One Health stresses the interdependence of human, animal, and ecosystem wellbeing, the Exposome provides its quantitative corollary, an attempt to measure that interconnectedness across time, space, and biological scale. The Exposome captures the totality of chemical, physical, biological, and even social exposures experienced from conception onward (18–21).

Epidemiological and population-cohort evidence indicates that inherited genetic variation explains only a minor share of the risk of most common chronic diseases, whereas the larger share is attributable to environmental and behavioral factors (22, 169). The Human Genome Project illuminated genetic code; the Human Exposome Project seeks to illuminate *context*. The distinction parallels that between traditional toxicology and the One Health approach: both require integration of mechanistic biology with ecological and social determinants.

### 1.3 One Health toxicology as a bridge between humans, animals, and ecosystems

The One Health paradigm emerged from the growing recognition that the health of humans, animals, and ecosystems is inseparable - a continuum rather than a hierarchy. The World Health Organization (23) defines One Health as “*an integrated, unifying approach that aims to sustainably balance and optimize the health of people, animals, and ecosystems*.” (7, 24). While the approach originated as a collaborative model for preventing zoonotic disease transmission-bridging human medicine, veterinary science, and ecology, it has since evolved into a broader epistemological framework encompassing chemical exposures, environmental pollutants, and toxicological stressors that traverse species boundaries and ecological compartments (25, 26, 166).

Historically, the field of toxicology was largely anthropocentric, focusing on direct human risk and employing animal models as proxies for human hazard assessment. However, the escalation of industrial chemical releases, agricultural runoff, pharmaceutical residues, and climate-induced distribution of pollutants has blurred distinctions between occupational, environmental, and ecological toxicology. As pollutants cycle through water, air, soil, and food webs, the fates of species become chemically coupled. One Health toxicology thus redefines chemicals not merely as agents of individual toxicity but as shared environmental stressors that generate networked risk across biological systems and geographies. Recent research reveals that exposomics (20) are expanding rapidly and that multi-stress environmental exposures across air, water, and soil are a central focus of contemporary environmental science and toxicology literature, reflecting real-world complexity rather than isolated toxicants (27–30).

At its core, One Health toxicology seeks to integrate multi-species surveillance, mechanistic toxicology, and ecological modeling into a unified risk-assessment architecture. The traditional focus on single-species dose–response relationships is replaced by an appreciation of interconnected exposure pathways-for example, how the accumulation of persistent organic pollutants in soils and sediments cycles through trophic systems to influence wildlife, domestic animals, and human populations. Crucially, the Exposome concept, i.e., the totality of an individual’s environmental exposures over a lifetime, is now recognized as essential to linking external chemical exposures with internal biological responses, and it is increasingly integrated into One Health toxicology frameworks (31). Surveillance data from wildlife, livestock, and human populations therefore become complementary components within a single continuum of evidence, transforming fragmented datasets into early warning systems for global environmental health. In parallel, interdisciplinary reviews illustrate how legacy pollutants such as dioxins exemplify the need for integrated One Health approaches, as these contaminants biomagnify through food webs, impact animal and human health, and require cross-sectoral monitoring strategies (32).

Recent scientific and regulatory initiatives drew attention to this transformation. The European Commission and the U.S. Environmental Protection Agency (33–35, 166) have begun to incorporate One Health principles into their chemical evaluation frameworks, emphasizing that toxicity testing, ecological impact assessment, and human health evaluation should be cross-referenced rather than sequential. Similarly, the growing movement for “*One Toxicology*” (26, 36) calls for dismantling disciplinary silos and promoting data interoperability between human toxicology, veterinary pharmacology, and ecotoxicology (37). The One Health framework thus provides not only a scientific rationale but also a policy mandate for integrating chemical risk governance across sectors.

The evolution of this concept parallels broader shifts in public health and Exposome thinking. The same systems-level insights that motivated One Health in infectious disease-recognition of complex feedback loops between species, environments, and societies-apply equally to chemical hazards. In this sense, One Health toxicology represents the chemical dimension of planetary health (8, 38), uniting the molecular mechanisms of toxicity with ecological feedbacks and social determinants of exposure. It aspires to replace the reactive model of crisis management (e.g., responding to poisoning outbreaks or contamination events) with predictive prevention, using shared biological and environmental indicators to forecast risk before harm occurs.

#### 1.3.1 Toward an integrated One Health toxicology

Collectively, these examples illustrate that toxicology can no longer be confined within species-specific or sector-specific boundaries. The One Health framework offers a conceptual and operational scaffold for integration across molecular, organismal, and ecosystem levels. It reframes chemical risk as a shared outcome of interconnected systems rather than an isolated property of individual compounds.

In practical terms, One Health toxicology entails:

- Establishing joint biomonitoring networks linking wildlife pathology, veterinary pharmacovigilance, and human public-health data.
- Using AOPs to mechanistically bridge effects observed across species.
- Embedding exposomics and systems toxicology into environmental risk assessment to capture cumulative and mixed exposures.
- Developing One Health-based risk communication that engages communities, regulators, and industries in co-managing environmental health.

Projects like Exposim and centralized exposomic data resources are emerging that aim to standardize, integrate, and make accessible large-scale exposure data for environmental and health research, demonstrating concrete steps toward integrated One Health toxicology (39, 40).^2^ Integrating these elements, toxicology evolves from a reactive science of damage control to a predictive science of prevention-one capable of safeguarding the health of all species that share an environment.

In short, One Health toxicology is not a niche subdiscipline but the next logical stage in the evolution of toxicology itself, aligning scientific rigor with ecological reality and ethical responsibility.

### 1.4 Why artificial intelligence and digital health technologies are needed

The One Exposome generates data that are too heterogeneous, high-dimensional, and dynamic to be interpreted through conventional analytical approaches alone. MPS, organoids, omics, biomonitoring, environmental sensors, geospatial monitoring, wearable devices, and citizen-generated data produce continuous streams of molecular, physiological, ecological, and contextual information. Artificial intelligence, in combination with mechanistic models, is therefore needed as the integrative layer that harmonizes fragmented data, detects patterns, and supports exposure–response prediction and actionable public health intelligence. At the same time, digital health technologies provide the operational infrastructure for real-time exposure capture, interoperable data exchange, sentinel surveillance, and decision-support dashboards. Together, AI and digital technologies enable the One Exposome to move beyond retrospective risk assessment toward predictive, preventive, and participatory environmental health governance.

### 1.5 Aim and scope of this contribution

Chemicals, drugs, and pollutants do not affect humans, animals, or the environment in isolation; they are all connected and the One Health approach recognizes this interdependence. This article explains how the One Health approach, which links human, animal, and ecosystem wellbeing, can be expanded to include toxicology. Using real examples, such as the die-off of vultures from veterinary diclofenac, mercury poisoning in Minamata, antibiotic resistance, and other complex multi-stressor exposures, the paper shows how chemical risks move through food webs and societies.

New scientific tools called NAMs, including organ-on-chip systems, artificial intelligence, and omics technologies, can replace many animal tests with the potential to provide faster and, in many contexts, more human-relevant predictions of harm. When combined with the Human Exposome Project, which integrates all environmental exposures over a lifetime, these tools form the basis of a new “*One Exposome*” framework. This integrated approach aims to predict and prevent chemical hazards, i.e., protecting people, animals, and the planet together and provides a more comprehensive understanding of the complex interactions between humans, animals, and the environment.

## 2 From the Human Exposome to the One Exposome

### 2.1 The Human Exposome: strengths and current limitations

The Exposome paradigm emerged as a “*life-course*” complement to genomics, linking environment, behavior, and internal physiology, precisely the domains unified under One Health. As articulated in the Johns Hopkins Exposome Collaborative framework (20) environmental exposure data, biofluids, and omics analyses are integrated to reveal signatures of external stressors and internal biological responses.

Like One Health, the HEP transcends disciplinary boundaries:

- *Environmental dimension:* monitoring of air, soil, water, food, and microbiomes, extending to eco-exposomes affecting animals and ecosystems.
- *Biological dimension*: human biomonitoring and omics for exposure fingerprints, mirroring One Health’s focus on shared molecular pathways across species.
- *Societal dimension:* inclusion of social stressors, occupation, and inequality as exposure modifiers, linking environmental justice to global health.

The HEP thus operationalizes One Health through measurement: where One Health is normative (“health is shared”), the Exposome is quantitative (“exposure is shared”).

### 2.2 One Health and toxicology: connecting human, animal, and environmental exposures

Systematic reviews reveal a consistent set of One Health toxicology archetypes that illustrate how toxicants transcend species and regulatory domains. Classic examples include neurological symptoms in cats from Minamata Bay that foreshadowed human methylmercury poisoning (13) and the vulture die-off in India caused by diclofenac (1). Sentinel organisms such as sea lions and dolphins have been proposed as indicators of endocrine and immunotoxic effects of persistent organic pollutants relevant to human populations (73, 74). Reviews and case studies (15, 41–43, 167 reveal a consistent set of One Health toxicology archetypes that illustrate how toxicants transcend species and regulatory domains. These recurrent patterns highlight both the interconnected nature of chemical hazards and the institutional gaps that amplify their consequences.

Recent Exposome research further supports this interspecies connection: studies highlight that internal exposomic signatures, reflecting combined influences of diet, pollutants, microbiome, and metabolism, may serve as biomarkers shared across human and animal health outcomes (38).

### 2.3 Defining the One Exposome

The *One Exposome* embodies the unification of *One Health*, *exposomics*, and *NAMs* into a single framework linking environmental exposures to biological responses across species and ecosystems (Figure 2). It reflects the realization that human, animal, and planetary health cannot be disentangled as their exposures are shared, their responses interconnected, and their solutions collective.

**Figure 2:**
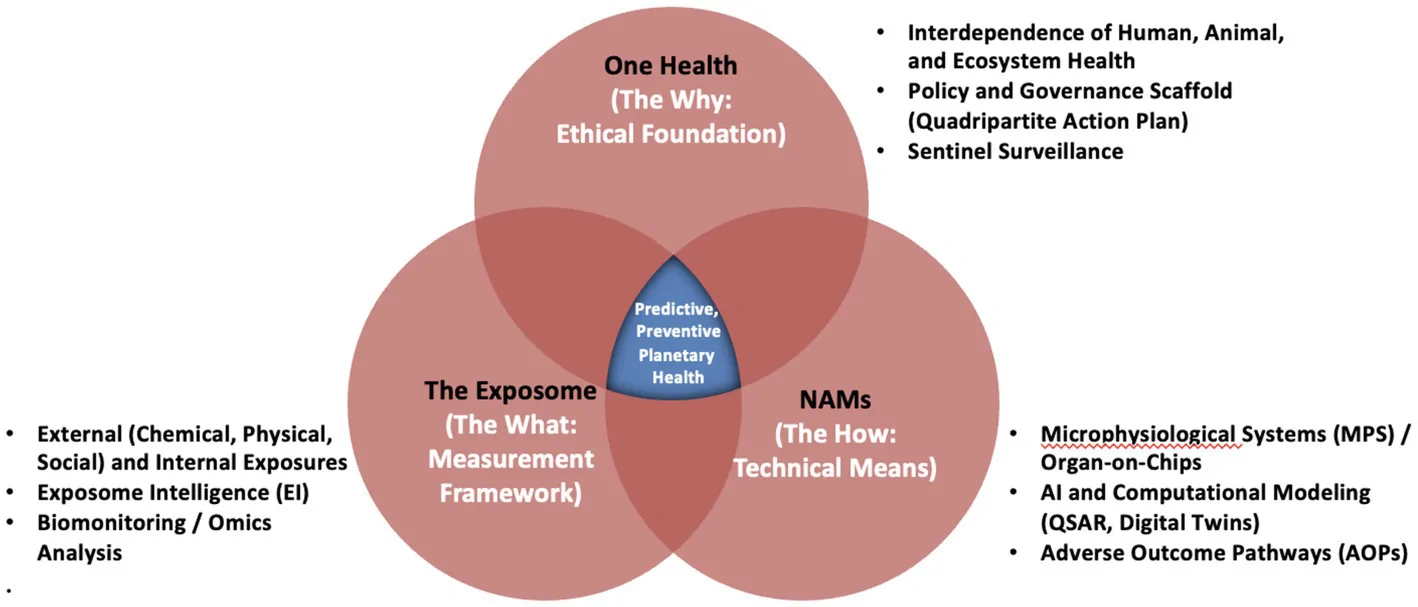
The One Exposome Paradigm. The diagram illustrates the intersection of three core elements: One Health (ethical/policy foundation), Exposome (quantitative/measurement framework), and NAMs (scientific/mechanistic backbone). Their overlap defines “*One Exposome*”.

As articulated in Sillé et al. (20), the exposome extends risk assessment beyond chemical-by-chemical testing to the totality of exposures over the life course. The *Human Exposome Project (HEP)* now transforms this conceptual breadth into an actionable global program, coupling the ethics of One Health with the analytics of Big Data. The unifying idea of One Exposome is therefore both epistemological and operational: to build a single evidence architecture for understanding how the environment shapes health, grounded in mechanistic human biology and supported by predictive computation.

One Health provided the ethical and ecological foundation, i.e., the recognition that zoonotic disease, antimicrobial resistance, and environmental degradation share common causes and therefore common remedies. Yet its implementation has remained largely qualitative.

The Exposome quantifies this interconnectedness. It provides the *measurement language* of One Health, the metrics by which shared risks and interventions can be evaluated. Within the One Exposome, the determinants of health are not partitioned into human, animal, and ecosystem compartments but treated as a continuous field of exposures and responses.

The transition from *One Health* to *One Exposome* thus parallels the shift from *genomics* to *exposomics*: from the static code of life to the dynamic context of living.

What is conceptually new. The One Exposome should not be read as yet another Exposome program competing with the Human Exposome Project (HEP), nor as a rebranding of One Health, but as an integrative conceptual architecture that binds three previously separate movements into a single operational logic. One Health established the normative claim that human, animal, and ecosystem health are interdependent, but has remained largely qualitative and infectious-disease oriented. The Exposome, and its flagship HEP, supplied the quantitative language of cumulative, life-course exposure, yet has concentrated on the human compartment and has largely excluded ecotoxicology and cross-species read-across. New Approach Methodologies (NAMs) provided human-relevant mechanistic evidence, but were developed chiefly for regulatory hazard assessment rather than for population-scale exposure science. The distinctive contribution of the One Exposome is therefore not the invention of a new data type but the explicit coupling of these three: it extends the Exposome across species and ecological compartments (going beyond the human-centred HEP), it equips One Health with a measurable, mechanistic substrate (going beyond qualitative One Health), and it embeds NAMs and AI as the analytic layer that renders cross-scale integration tractable. In this respect it is consistent with, and complementary to, the current trajectory of environmental-health research toward systems-based, implementation-oriented frameworks such as Safe-and-Sustainable-by-Design (SSbD) and Next Generation Risk Assessment (NGRA) (44, 45), and with emerging work on AI-assisted Exposome interpretation, mechanistic exposure-response modeling, and Exposome-enabled decision support (46–49). The inclusion of New Approach Methodologies (NAMs) have provided human-relevant mechanistic and design evidence, but they should be considered beyond their traditional mechanistic value. NAMs can be integrated from the outset in Stage 1 of the workflow, contributing to data collection and harmonization across various domains, including emerging new One Health areas. Crucially, the One Exposome is offered as a conceptual and organizing framework rather than as a new independent infrastructure or funding program; its purpose is to align existing initiatives (One Health, the HEP, NAM validation efforts, and AI and digital-health platforms) around a shared object of study: the totality of shared exposures and responses across the human/animal/environment continuum. By considering NAMs from the very beginning of the workflow, we can leverage their full potential across all aspects of One Health, integrating methods and models to address diverse health challenges.

#### 2.3.1 An operational workflow for the One Exposome

To make the framework actionable rather than aspirational, it is useful to specify how heterogeneous data would be integrated, by whom, and toward what decision (Figure 3). In the proposed workflow, (i) data producers (environmental-monitoring agencies, human biomonitoring and clinical cohorts, veterinary and wildlife surveillance networks, and citizen and wearable streams) generate primary observations; (ii) these are harmonized through FAIR (Findable, Accessible, Interoperable, Reusable) data stewardship and shared ontologies into an interoperable One Exposome data commons; (iii) NAMs, organ-on-chip systems, and omics provide mechanistic, human-relevant anchoring, while machine-learning and knowledge-graph methods perform exposure reconstruction, mixture and cross-species inference, and pattern detection; (iv) outputs are passed through an Evidence-to-Decision (EtD) layer (50) using explainable, human-supervised AI that weighs benefit, harm, equity, and feasibility; and (v) the resulting evidence products support regulators, public-health authorities, and communities in prioritization, early warning, and prevention. Responsibility is distributed but explicit: data stewards and repositories curate and govern the commons; NAM and AI developers validate and document their models (51); domain scientists interpret; regulatory and public-health bodies own the final decisions; and community advisory boards retain oversight of data use. We present this workflow as a testable operating model rather than a finished system, noting that each hand-off defines a concrete locus for validation, accountability, and quality control.

**Figure 3:**
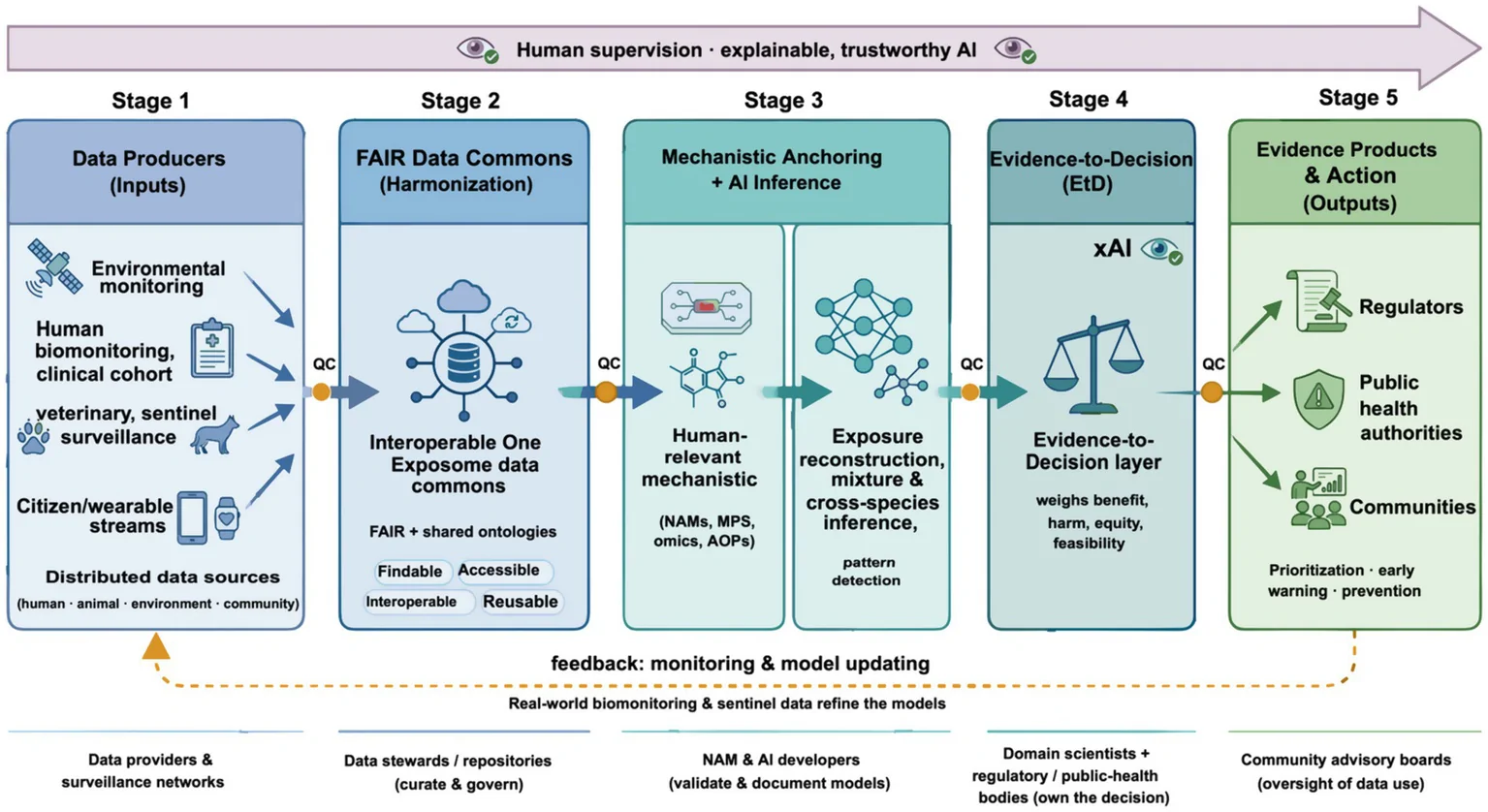
A proposed operational workflow for the One Exposome. Heterogeneous data from environmental monitoring, human biomonitoring and clinical cohorts, veterinary and wildlife sentinel surveillance, citizen and wearable streams, and New Approach Methodologies (NAMs), including method and model development (Stage 1), are harmonized through FAIR stewardship and shared ontologies into an interoperable data commons (Stage 2). Microphysiological systems and omics provide human-relevant anchoring, while machine-learning and knowledge-graph methods perform exposure reconstruction and cross-species inference (Stage 3). Outputs pass through an Evidence-to-Decision layer using explainable, human-supervised AI (Stage 4) to generate evidence products that support regulators, public-health authorities, and communities in prioritization, early warning, and prevention (Stage 5). A feedback loop returns real-world biomonitoring and sentinel data to refine the models; responsibility is distributed and explicit, and each hand-off constitutes a checkpoint for validation, accountability, and quality control.

### 2.4 Key exposure domains: chemical, biological, physical, social, and ecological stressors

Recent evidence highlights that pollutants in air, water, and soil, such as fine particulate matter, per- and polyfluoroalkyl substances (PFAS), microplastics, heavy metals, and chemical mixtures, contribute significantly to human and ecosystem chemical exposomes and interact with biological systems via overlapping mechanistic pathways (31, 52, 53). For example, air pollution as a domain contributes to systemic inflammatory and oxidative stress pathways that are mechanistically linked to health outcomes across species (53). Similarly, exposome research highlights how chemical mixtures, such as PFAS, pesticides, and heavy metals are detected in human biomonitoring and linked to mechanistic changes in metabolism, immune signaling, and organ systems (31, 54).

The plastisphere, i.e., the ecological community of microorganisms and other small organisms that live on and around plastic debris in natural environments, especially in the ocean, is an engine of environmental change; it can have significant impacts on biogeochemical cycling and pollutant fate, and the occurrence and impacts of environmental contaminants, including their bioaccumulation, biomagnification, and acute, subacute, and chronic effects on organisms and ecosystems, must be addressed through innovative research and evidence-based policymaking and connecting transdisciplinary areas. Addressing such cross-compartment challenges also requires shared, interoperable data infrastructures. The European Commission has promoted the development of common European Data Spaces in several macro areas of public interest, including Manufacturing, Green Deal, Mobility, Finance, Energy, Agriculture, Public Administration, Skills, and Health (55, 165). The European Health Data Space (EHDS)^3^ is an exemplary case among the 14 Common European Data Spaces currently under development, with strategic goals that include making data available to support prevention, detection, and treatment strategies, as well as to inform evidence-based decisions to improve the healthcare system (56).

The One Health concept highlights the inextricable link between human, animal, and ecosystem health, an essential perspective for addressing environmental pollution. Industrial activities, agriculture, and urbanization have introduced a multitude of contaminants into the air, water, and soil, threatening ecosystems and posing significant risks to human and animal health. The use of NAMs is a key component of the One Health approach, enabling the development of more effective and sustainable solutions to health challenges. NAMs include cell and tissue methods (*in vitro*), structure-based/toxicokinetic models (*in silico*), methods that assess toxicant interactions with biological macromolecules (*in chemico*), and alternative models. Integration with multi-omics exposomics and AI now allows comprehensive mapping from external chemical exposures to mechanistic biological outcomes, bridging gaps between environmental contamination and human health (52, 54).

The integration of NAMs into such One Health evidence-based decision-making requires a standardized framework for the application of NAMs, including the use of QIVIVE and the consideration of toxicokinetics (57, 58) as well as toxicodynamics. This framework should be flexible and adaptable to different regulatory contexts and should include the use of uncertainty factors to account for variability and uncertainty in NAMs data (12). The One Health approach compels a paradigm shift from this fragmented model toward a transdisciplinary, systems-based toxicology. It calls for surveillance networks that integrate wildlife morbidity data as early indicators of environmental contamination, for mechanistic frameworks like adverse outcome pathways (AOPs) that enable cross-species extrapolation, and for regulatory structures capable of considering population- and ecosystem-level effects. This paradigm also explicitly incorporates real-world chemical mixtures and emerging environmental stressors within predictive and preventive frameworks (59, 60). The diclofenac disaster underscores that the boundaries between pharmacology, toxicology, and ecology are artificial. Drugs, like pollutants, move through biological networks that link the fates of animals, humans, and the environment. Understanding and mitigating such interconnections is no longer optional, it is central to safeguarding global health.

## 3 Data foundations for AI-enabled One Exposome research

### 3.1 Human biomonitoring, epidemiological, and clinical data

NAMs provide the scientific backbone for measuring, modeling, and mitigating the Exposome. Their integration marks the transition of toxicology into a predictive, human-relevant science (61). Together, NAMs convert descriptive exposure science into a mechanistic systems biology of health.

The HEP operationalizes the One Exposome vision. It combines biomonitoring, untargeted metabolomics, MPS-based mechanistic testing, and AI-driven analytics into a closed evidence loop. Each component feeds the other:

- Epidemiological data generate exposure/disease associations.
- Computational models distill these into causal hypotheses.
- MPS models test these hypotheses under human-relevant conditions.
- Mechanistic findings refine exposure models and biomarkers, which feed back into population studies.

This recursive, data-driven architecture embodies One Exposome in action, a learning system that continuously aligns molecular biology with public health.

The Implementation Moonshot Project (IMPACT) was launched to operationalize the HEP by integrating MPS, omics, AI, and real-world exposure data (62). It converged with the Exposome Moonshot Forum (63–65), producing the *Washington Declaration on the Human Exposome* and establishing a Global Exposome Consortium^4^.

Parallel initiatives, e.g., the NIEHS NEXUS^5^ network, the European Human Exposome Network (EHEN)^6^, and the EIRENE^7^ research infrastructure, are aligning under a new International Human Exposome Network (IHEN)^8^ in Europe and globally as the Global Exposome Forum^9^, to standardize data and ethics governance. Together these efforts echo the transatlantic cooperation that defined the Human Genome Project, extending it now to the environmental dimension of health.

### 3.2 Environmental monitoring and geospatial exposure data

The eco-exposome concept expands the Human Exposome to include the shared environmental matrices that determine cross-species exposure, air, water, soil, and microbiomes (20, 66, 67). Building on One Health surveillance, integrating eco-exposomes entails coupling environmental monitoring with wildlife sentinels and human biomonitoring in unified digital platforms.

Such integration demands harmonized sampling protocols, interoperable metadata standards, and transdisciplinary analytics linking molecular biomarkers with ecological indicators. For example, high-resolution mass-spectroscopy profiles from aquatic organisms could be aligned with human metabolomics and remote-sensing pollution data to map chemical gradients across ecosystems (68, 69). Progress in mass spectrometry (70–72) is very much accelerating these developments. Eco-exposome (38, 67) integration thus operationalizes One Health’s core premise, that exposures are shared and that protecting any species requires protecting all.

### 3.3 Veterinary, wildlife, and sentinel-species data

One Health toxicology extends the classical four-step risk assessment (i.e., hazard identification, dose–response assessment, exposure assessment, and risk characterization) into a multi-directional, cross-species surveillance framework (166). This expansion acknowledges that early warnings of chemical hazard often emerge first in wildlife or domestic animals long before manifesting in humans.

Sentinel species such as raptors, amphibians, and marine mammals serve as biological barometers of environmental health. Their physiology, lifespan, and ecological niche make them ideal integrators of exposure signals. For example, mercury-induced neurological dysfunction in fish-eating birds, immunotoxicity in marine mammals, or reproductive anomalies in amphibians have been proposed as sentinel events for equivalent pathologies in human populations (43, 73). Integrating such sentinel data with environmental monitoring transforms ecological observation into a predictive public health tool, a feedback system capable of foreseeing harm before it reaches human communities.

This synergy extends beyond the laboratory. Integrated NAM–AI systems can support sentinel surveillance for environmental and veterinary health, detecting signals of ecological perturbation (e.g., pollution-induced endocrine disruption) that foreshadow human health risks. Linking environmental biosensor networks, wildlife sentinel data (73, 74), and AI-driven exposomic analytics operationalizes One Health at the systems level (62, 75).

### 3.4 Omics, toxicogenomics, and molecular biomarkers

Systems toxicology represents the mechanistic fusion of biology, computation, and chemistry, an approach that moves beyond isolated biomarkers to model entire regulatory networks perturbed by environmental stressors (76, 77). By combining multi-omics technologies (transcriptomics, proteomics, metabolomics, and epigenomics) with advanced analytics, systems toxicology maps conserved molecular pathways of injury across species.

The exposome framework, first articulated by Wild (21), complements this approach by quantifying the totality of exposures an organism experiences throughout life, chemical, physical, biological, and social (22). Integrating systems toxicology with exposomics allows direct linkage between external environmental inputs and internal molecular responses. The result is a causal continuum from pollutant emission to gene regulation, phenotype alteration, and disease.

Practically, this integration is realized through MPS, human-relevant organoids and organs-on-chips that replicate organ-level physiology under controlled conditions (71, 78). When combined with omics readouts, these systems yield mechanistic fingerprints that feed into AI-driven exposome analytics (79). Machine learning and causal-inference models can detect hidden exposure/effect relationships across species, chemicals, and time scales, transforming large, heterogeneous datasets into predictive maps of chemical risk.

Systems toxicology thus forms the analytical cortex of the One Health Intelligence System envisioned in Section 5. By modeling molecular networks as dynamic systems influenced by multiple stressors, it enables early identification of tipping points where compensatory mechanisms fail and pathology emerges. Importantly, these insights are not confined to humans: the same oxidative-stress, inflammation, and endocrine pathways recur across vertebrate species, allowing translation from *in vitro* human data to ecological risk contexts.

In the regulatory domain, coupling systems toxicology and exposomics supports the shift toward mechanistic, NAM-based evaluation. Data from multi-omics MPS and *in silico* modeling can replace many animal studies by providing pathway-level evidence directly linked to AOP frameworks (80, 81). Furthermore, real-world exposome monitoring, including wearable sensors and environmental biosensors, can validate these models under authentic exposure conditions (79). Together, these advances forge a quantitative bridge between laboratory prediction and environmental reality.

### 3.5 New approach methodologies, organoids, and microphysiological systems

#### 3.5.1 NAMs as the scientific infrastructure of One Health

This section outlines how NAMs, particularly microphysiological systems (MPS) (82–84) and AI-driven models (61, 68, 75, 85, 156), enable predictive, human-relevant toxicology aligned with One Health objectives. It explores how mechanistic modeling, data-driven learning, and human-derived test systems can transform global chemical safety evaluation into a sustainable, preventive science.

The One Health concept calls for systems approaches to complex health challenges spanning species and environmental compartments. NAMs operationalize this philosophy in toxicology (12, 155). Rather than using surrogate animal models to infer human risk, NAMs rely on human-derived cells, computational models, and mechanistic AOP frameworks to map causal chains across biological levels.

In One Health toxicology, NAMs serve three converging functions:

1. *Mechanistic integration:* linking molecular initiating events to organismal and ecological outcomes via AOP networks.
2. *Cross-species extrapolation:* leveraging conserved pathways to assess shared susceptibility across humans, wildlife, and domestic species.
3. *Predictive prevention:* using high-content, AI-supported modeling to anticipate toxicological cascades before they manifest as crises.

NAMs thus provide the mechanistic glue between environmental monitoring, veterinary pharmacology, and human safety science, fields historically separated by methodology and regulation.

#### 3.5.2 MPS, the human-equivalent test bed

Among NAMs, MPS – which encompass a range of engineered human-relevant models, including organ-on-chip and multi-organ-chip systems, and may incorporate organoids or other three-dimensional tissues offer the most tangible bridge between reductionist cell assays and whole-organism complexity. These systems bioengineer human organ functionality at micro-scale by combining induced pluripotent stem cell (iPSC)-derived tissues with microfluidic perfusion, enabling dynamic modeling of physiological conditions (78).

#### 3.5.3 Mechanistic fidelity and human relevance

MPS replicate essential aspects of organ physiology, e.g., fluid shear stress, barrier integrity, immune competence, and metabolic coupling, allowing modeling of organ-level toxicities, systemic pharmacokinetics, and chronic exposures. Liver-, kidney-, lung-, brain-, and cardiac-on-chip models have shown promising – and in selected applications improved – human-relevant prediction of drug-induced liver injury, neurotoxicity, nephrotoxicity, and cardiotoxicity (86, 87).

Crucially, MPS address One Health’s mechanistic imperative: they permit direct investigation of shared toxicity pathways across species and environmental exposures. For example, a lung-on-chip can simulate respiratory responses to particulate matter relevant for both occupational human exposures and wildlife ecotoxicology, while brain organoids inform developmental neurotoxicity in humans, neurological diseases such as autism and Alzheimer’s disease and ecological sentinels alike.

#### 3.5.4 Validation, standardization, and regulatory progress

The establishment of international consortia, such as the International MPS Society (iMPSS)^10^ and the MPS World Summits,^11^ has accelerated the harmonization of terminology, quality standards (Good Cell and Tissue Culture Practice 2.0; (80, 88)),^12^ reporting standards (GIVReSt, (89)) and validation frameworks (51). The FDA Modernization Act 2.0 (2022) and NIH’s Complement-ARIE^13^ initiative now permit and actively support the use and validation of MPS and other NAMs as nonclinical evidence in drug safety evaluation (68, 90, 91).

Independent validation centers, reference compound libraries, and tiered qualification pathways are under development, paving the way for accelerated regulatory acceptance in pharmacology, chemical safety, and environmental risk assessment (92) valorizing *in silico* process flows, AI and digital health technologies.

#### 3.5.5 MPS and ecosystem toxicology

Although MPS were initially conceived for human health applications, their potential extends into ecotoxicology and planetary health. Species-specific MPS (e.g., fish liver or avian kidney-on-chip models) could provide mechanistic anchors for wildlife hazard evaluation, linking laboratory results to field observations such as the diclofenac–vulture collapse. If developed and validated, such cross-species MPS models could offer a new level of resolution to predict, or rapidly verify, hazards before they reach the ecosystem scale.

### 3.6 NAMs as the engine of Exposome science

#### 3.6.1 From hazard to mechanism

The HEP cannot rely on legacy animal testing, which is too slow, coarse, and species-restricted to capture mixture or low-dose effects. Instead, NAMs, *in vitro*, *in silico*, and *in chemico* systems, provide mechanistic resolution at human scale (93, 161, 163). As emphasized in ToxAIcology (61) and Hartung and Smirnova (78), these technologies convert the traditional risk equation into a data-driven, predictive science.

NAMs deliver the experimental substrate of the HEP by:

- Mapping AOPs that anchor omics signatures to disease mechanisms;
- Enabling cross-species extrapolation within a One Health framework; and
- Providing reproducible, ethical, human-relevant evidence for exposure–effect relationships.

#### 3.6.2 Microphysiological systems (MPS): human biology in miniature

MPS, mainly organoids and organs-on-chips, are the physical test-beds of exposome research (71). They replicate tissue–tissue interfaces, perfusion, and metabolism, allowing direct observation of pollutant, dietary, or pharmaceutical exposures at realistic concentrations.

Figure 4 illustrates this workflow: biofluids analyzed by omics yield exposure hypotheses, which are then tested in MPS to map perturbed pathways and generate *digital twins* of organ responses (71). These systems permit mechanistic anchoring of statistical exposome associations, fulfilling the One Health call for causal understanding across species and environments.

**Figure 4:**
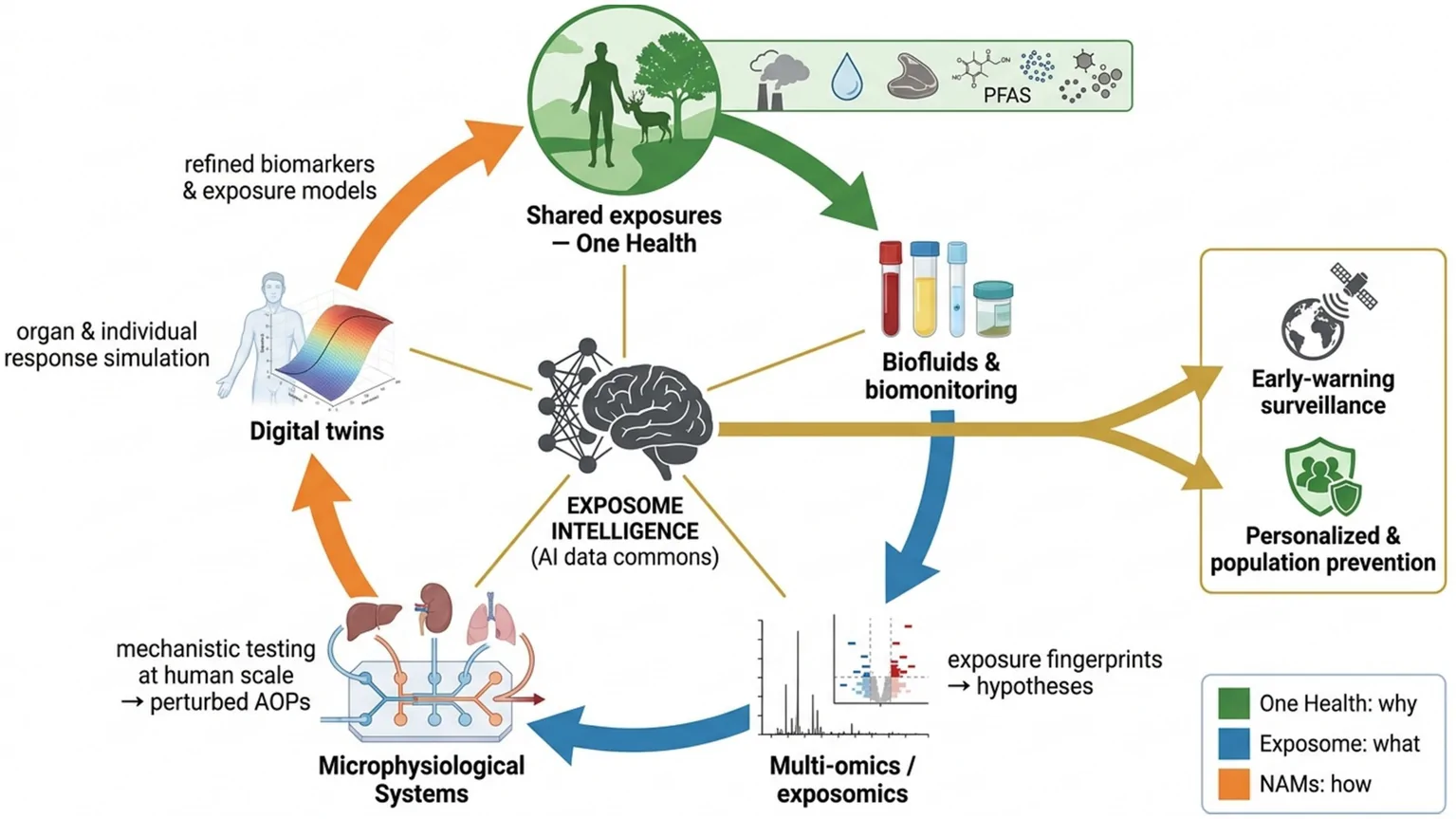
The One Exposome closed evidence loop (conceptual architecture). Shared exposures across humans, animals, and ecosystems (green, One Health) are captured through biofluids and biomonitoring and resolved by multi-omics exposomics (blue, exposome) into exposure fingerprints and hypotheses. These are tested mechanistically in MPS systems (organoids, organs-on-chips) and propagated into digital twins of organ and individual responses (orange, NAMs), mapping perturbed AOPs at human scale. Refined biomarkers and exposure models feed back into population studies, closing a recursive, self-correcting loop. At its centre, Exposome Intelligence, a proposed AI-driven data commons, would integrate all streams (radial connectors) and drives the two principal outputs of the system: Early-warning surveillance and personalized and population-level prevention. The colour code (green/blue/orange) maps to the One Exposome triad: One Health (*why*), exposome (*what*), and NAMs (*how*).

### 3.7 FAIR data, interoperability, and data-quality challenges

#### 3.7.1 Data heterogeneity and interoperability

The most immediate challenge is data heterogeneity, the integration of molecular, environmental, and social datasets produced at vastly different temporal, spatial, and analytical scales (50, 160). Exposome data originate from sources as diverse as satellite-derived pollution metrics, wearable-sensor streams, untargeted metabolomics, high-throughput screening, and socio-economic registries. Each domain employs distinct ontologies, metadata structures, and privacy regimes, impeding cross-domain synthesis (20, 79).

To transform this patchwork into a coherent evidence ecosystem, syntactic, semantic, and ethical interoperability must evolve in parallel. Technically, this requires adoption of FAIR^14^ data principles, standardized identifiers for chemicals and biomarkers, and machine-readable metadata schemas (94, 95) that connect laboratory-generated mechanistic data with large-scale epidemiological observations. Ontology frameworks such as the OECD AOP-Wiki,^15^ BioBricks registry (96), and NIEHS NEXUS^5^ data commons already provide templates for harmonization but must be extended to encompass behavioral and ecological variables.

Equally critical is data quality assurance. The reproducibility of exposomic associations depends on transparent documentation of sample provenance, analytical uncertainty, and contextual metadata. Establishing confidence tiers for exposome datasets-analogous to evidence grading in evidence-based toxicology-will allow regulators to weigh heterogeneous inputs proportionally. Moreover, joint environmental and biological monitoring, from sentinel species to air, water, and soil sensors, forms early warning systems capable of detecting emergent chemical, microbial, and physical hazards before they manifest in humans or ecosystems.

The ultimate goal is to evolve from fragmented “*data lakes*” to federated, query-ready exposome networks, where algorithms can learn across distributed datasets without violating privacy or data sovereignty (79).

#### 3.7.2 Standardization without stagnation

A second barrier lies in achieving standardization without stagnation, e.g., the tension between harmonizing methodologies and preserving scientific innovation and accelerated decision-making. Excessive procedural rigidity risks freezing progress; insufficient standardization undermines comparability.

The solution is adaptive but accelerated standardization: modular quality systems that define minimum essential criteria while allowing iterative refinement. Frameworks such as Good Cell and Tissue Culture Practice 2.0 (GCCP 2.0) and Good *In Vitro* Reporting Standards (GIVReSt) (80, 89) exemplify this balance. They mandate transparency, traceability, and reproducibility without prescribing specific technologies, thereby enabling continuous innovation in MPS, organoid, and AI workflows.

Cross-sector collaboration is vital. The International MPS Society (iMPSS),^16^ Exposome Moonshot Forum,^17^ and OECD AOP^18^ development program have each demonstrated that standardization is most successful when codesigned by regulators, industry, and academia. Shared reference compounds, inter-laboratory ring trials, and open benchmarking datasets provide empirical anchors for method comparison.

Finally, computational reproducibility and scientific relevance must complement laboratory harmonization. Version-controlled pipelines, open-source algorithms, and continuous-integration testing will ensure that computational exposomics achieves the same rigor expected of wet-lab experiments (68, 97). Our proposal for Evidence-based AI aims to bring this rigor to AI (98).

## 4 Artificial intelligence methods and digital health technologies for the One Exposome

### 4.1 Machine learning for exposure prediction and reconstruction

As exposomics and MPS produce terabytes of multi-modal data, AI becomes the analytical cortex. Deep learning and causal-inference algorithms unify chemical, omics, geospatial, and behavioral data into coherent exposure–effect models (79). AI also powers “*e-validation*,” i.e., dynamic, transparent benchmarking of NAMs, (92, 99) and supports *digital-twin* simulations that predict risk trajectories for individuals or populations.

In Hartung (79), this fusion is termed Exposome Intelligence (EI): a proposed privacy-preserving data commons that would integrate wearables, clinical cohorts, and citizen science to enable real-time exposure surveillance and individualized prevention. Within One Health, EI could function as a global early-warning system connecting human and environmental data streams.

### 4.2 AI for hazard identification and chemical prioritization

In the *ToxAIcology* framework, Hartung (61) we conceptualized AI as the “*new electricity*” of toxicology, turning *Big Data* into *Big Sense*. Machine learning, deep neural networks, and causal modeling tools can now decode complex biological patterns, predict toxicity across chemicals and species, and accelerate hypothesis generation for ToxAIcology beyond human capacity (100).

AI-driven quantitative structure–activity relationship (QSAR) models and graph neural networks have demonstrated strong predictive performance for selected toxicological endpoints, including mutagenicity and hepatotoxicity (101, 102). Integration of chemical structure, bioactivity, and omics data enables multi-modal toxicity forecasting and probabilistic risk assessment (103–105).

### 4.3 Read-across, structure–activity relationships, and predictive toxicology

Combining MPS experimental data with AI algorithms allows creation of digital twins, i.e., virtual representations of organ systems for *in silico* testing and scenario simulation (106). Such biological–digital integration enables proactive identification of chemical hazards and optimization of interventions under One Health constraints.

### 4.4 Knowledge graphs and causal AI for mechanistic integration

The real opportunity of NAMs lies in their convergence: MPS as data engines generating high-fidelity human biology, and AI as the analytical cortex transforming these data into actionable knowledge. Together, they enable a closed, learning loop of *experiment–prediction–validation* that mirrors living systems themselves.

By training AI models on MPS-derived datasets, it becomes possible to dynamically refine toxicity predictions, validate AOP hypotheses, and identify biomarkers in near real time. These iterative cycles constitute a “self-improving toxicology ecosystem” where each new experiment strengthens the model and each new model guides the next experiment.

### 4.5 Digital twins and simulation of exposure-response scenarios

Digital twins, i.e., computational surrogates trained on MPS and omics data, enable longitudinal modeling of exposure, susceptibility, and disease. By populating cohorts of such twins, the HEP could simulate population-level exposure scenarios while preserving individual variability (71). These digital cohorts bridge experimental toxicology with public-health modeling, creating a continuous evidence loop from molecule to population: the functional realization of One Health systems thinking.

Exposome Intelligence (EI) is proposed to integrate AI, cloud computing, and wearable sensors into a real-time health-surveillance network. It would allow *digital twins* to simulate exposures, predict outcomes, and personalize prevention strategies. As outlined by Hartung (79), such models could detect early warning signals from environmental sensors, social data, and physiological responses, ultimately creating a planetary early-warning system for health.

These digital ecosystems close the loop between observation, mechanism, and intervention, the same principle that underlies ecological homeostasis, now implemented through data science.

### 4.6 Explainable, trustworthy, and human-supervised AI

For regulatory science, transparency is paramount. Explainable AI (xAI) techniques, including perturbation-based feature attribution and surrogate models, now make it possible to visualize which molecular or cellular factors drive toxicity predictions, aligning AI outputs with mechanistic AOP frameworks (107, 108). This interpretability bridges the “*black box*” concern and supports integration of AI models into chemical risk assessment workflows, ensuring traceability, fairness, and reproducibility.

### 4.7 Digital health technologies

#### 4.7.1 Wearable and ambient sensor networks

The One Exposome requires continuous, real-time capture of external exposures across environmental compartments. Wearable biosensors (e.g., wristbands for VOC and UV exposure, portable spectrometers for air quality, ingestible sensors for internal biomarkers) and fixed environmental sensor networks (air quality monitors, water quality probes, soil contaminant sensors) can generate high-frequency exposure data streams. These technologies are already deployed in Human Exposome studies and could be extended into a One Health framework by linking human wearable data with co-located wildlife and environmental sensor readings.

#### 4.7.2 Mobile health, citizen science, and community-generated data

Validated digital health and citizen-science platforms can strengthen One Exposome surveillance by extending data collection beyond conventional monitoring systems. Smartphone apps, participatory epidemiology portals, and community-reporting tools enable citizens to contribute geolocated exposure observations, symptom reports, and environmental information, with particular value in low- and middle-income countries where fixed monitoring infrastructure is limited. Examples include citizen-science initiatives such as Safecast^19^ and iNaturalist.^20^ To ensure scientific utility, crowdsourced data require robust quality-assurance procedures, including standardized metadata, sensor calibration, validation against reference data where feasible, uncertainty reporting, and bias assessment. Because these data may contain geolocation, symptom, environmental, and other potentially sensitive health information, their use in public health decision-making also requires clear ethical safeguards, including informed consent, privacy protection, transparent data governance, community feedback, and fair benefit-sharing.

The European Health Data Space (EHDS)^21^ provides a regulatory and technical framework for secure access, exchange, and secondary use of electronic health data across the European Union. In the context of the One Exposome, it can serve as a model for broader data infrastructures linking human health, biomonitoring, environmental, veterinary, wildlife, and sentinel-species data. However, the EHDS alone is not sufficient: One Exposome research requires interoperability across health, environment, agriculture, food safety, toxicology, and biodiversity domains. Initiatives such as EIRENE, the NIEHS NEXUS network, and emerging exposome data commons illustrate the move toward federated, AI-ready infrastructures. Federated architectures are particularly relevant because they allow distributed analysis while preserving privacy, institutional control, and data sovereignty. Synthetic data may support testing and method development, but should not replace well-characterized empirical evidence. The central challenge is therefore not only technical interoperability, but also semantic, ethical, and governance interoperability. Together, these infrastructures can support Digital Health Intelligence by transforming heterogeneous exposome data into actionable knowledge for prediction, prevention, and environmental public health.

#### 4.7.3 Digital health integration platforms and interoperability architectures

Digital Health Integration Platforms provide the software infrastructure required to connect heterogeneous One Exposome data streams into usable public health intelligence. These platforms link health information systems, electronic health records, clinical decision-support tools, epidemiological surveillance systems, veterinary health databases, and environmental monitoring infrastructures through secure application programming interfaces (APIs). Their value lies in enabling timely data exchange across sectors that are usually managed separately, including human health, animal health, food safety, environmental monitoring, and chemical risk assessment. Interoperability standards such as HL7 FHIR,^22^ the OMOP Common Data Model,^23^ WHO Digital Documentation of COVID-19 Certificates,^24^ and OBO Foundry^25^ ontologies illustrate how structured data models and shared vocabularies can support cross-domain integration. Within a One Exposome framework, such architectures could support a One Exposome Dashboard: a visualization and decision-support interface that combines biomonitoring, environmental, sentinel-species, and toxicological data into actionable intelligence for policymakers. The goal is not simply data aggregation, but the transformation of distributed evidence into transparent, timely, and prevention-oriented decisions for environmental public health.

## 5 Applications in One Health toxicology and environmental public health

### 5.1 Chemical mixtures and cumulative risk assessment

Beyond single-chemical tracking, modern One Health surveillance addresses cumulative and interactive exposures. Organisms in the environment are rarely exposed to one compound at a time; rather, they encounter mixtures of metals, pesticides, pharmaceuticals, and microplastics that act synergistically or antagonistically. Cumulative risk assessment (CRA) frameworks now incorporate multiple chemical and non-chemical stressors, accounting for additive or non-linear interactions (104, 105).

Recent advances in high-throughput toxicokinetic (HTTK) modeling (57) and machine learning enable the integration of exposure data from diverse sources (soil, water, air, and food) into probabilistic, multi-pathway models (104, 109). These approaches quantify aggregate risk while explicitly modeling uncertainty, enabling policymakers to evaluate trade-offs in complex exposure scenarios.

Cumulative assessment also includes ecosystem services as endpoints. The loss of pollination, nutrient cycling, or water purification caused by chemical stress is not an ecological externality but a measurable degradation of human wellbeing. Embedding sentinel surveillance within ecosystem modeling thus unites environmental protection and human health under a single, data-driven framework, a practical expression of One Health risk analysis.

### 5.2 Pharmaceuticals, pesticides, and endocrine-disrupting chemicals

#### 5.2.1 Pharmaceutical ecotoxicity: the diclofenac disaster

The diclofenac case (Section 1.1) exemplifies cross-species pharmaceutical hazard. Widely used therapeutically in humans and livestock at approved doses, it triggers renal necrosis and visceral gout in vultures at ppm tissue levels (1). Post-market monitoring failures allowed its widespread use in Indian cattle in the 1990s, generating mass ecological mortality and public-health fallout (3, 4). The ban of veterinary diclofenac in 2006 and substitution with meloxicam, proven vulture-safe, highlighted the preventive potential of One Health risk evaluation.

#### 5.2.2 Heavy-metal and industrial contamination

The Minamata disease outbreak in mid-20th century Japan remains the quintessential One Health toxicology case study linking environmental pollution to cross-species toxicity. The release of methylmercury from industrial effluents into Minamata Bay caused bioaccumulation in fish and shellfish, which subsequently poisoned domestic animals and humans (13). Remarkably, neurological disorders first appeared in cats, exhibiting convulsions and erratic behavior (“dancing cat disease”) before human cases were clinically recognized. The episode epitomized the failure of unintegrated surveillance: ecological warning signs were visible but institutionally siloed, delaying intervention and amplifying human tragedy.

This environmental-to-human cascade underscores how aquatic ecosystems function as amplifiers and sentinels of industrial toxicants. Mercury methylation by anaerobic microorganisms transforms inorganic mercury into its far more bioavailable and neurotoxic form, creating persistent contamination that percolates through trophic levels. Similar pathways characterize the bioaccumulation of cadmium, lead, and arsenic, all of which exhibit species-transcending toxicodynamics, disrupting neurological, renal, and reproductive systems in both wildlife and humans (69).

Today, comparable cross-domain exposure patterns persist in artisanal and small-scale gold mining (ASGM) regions across Africa, Asia, and South America. Mercury is used to amalgamate gold from ore, often under open-air burning conditions, contaminating soils, waterways, and food chains. Livestock graze on mercury-laden vegetation, fish concentrate the metal in their tissues, and rural populations consuming these resources suffer cumulative neurotoxic and developmental impairments. The scale of this challenge is immense - an estimated 10–15 million artisanal miners and 100 million people are exposed worldwide (110).

Beyond mercury, industrial effluents rich in lead, chromium, and persistent organic pollutants (POPs) from tanneries, e-waste recycling, and smelting operations demonstrate that chemical pollutants respect neither regulatory nor species boundaries. For example, lead-acid battery recycling in informal sectors contaminates residential soils, simultaneously affecting children’s neurodevelopment, livestock growth, and local biodiversity. These intertwined risks reaffirm that industrial contamination is a shared ecological stressor requiring co-monitoring of sentinel species, human biomarkers, and environmental matrices.

Integrated mitigation strategies, such as bioindicator species monitoring, ecotoxicological modeling, and joint environmental–public health surveillance, are increasingly being adopted. Yet effective intervention still hinges on regulatory synchronization among environmental, labor, and health authorities. The Minamata Convention on Mercury (2013) represents an important global milestone, but its success depends on the degree to which countries implement One Health surveillance and community-based remediation programs to close the gap between international policy and local exposure realities.

#### 5.2.3 Food-chain mycotoxins

Mycotoxins illustrate the agro-ecological dimension of One Health toxicology, where microbial metabolites produced under environmental stress infiltrate both the food and feed chains. The best-studied example, aflatoxin B₁, produced by *Aspergillus flavus* and *A. parasiticus*, contaminates grains and oilseeds during pre-harvest drought or post-harvest storage under humid conditions. When ingested by dairy cattle, it is hydroxylated to aflatoxin M₁, which appears in milk and dairy products, creating a direct exposure pathway to humans (42, 111). Chronic intake contributes to hepatocellular carcinoma, immunosuppression, and child growth impairment, making aflatoxins among the most potent naturally occurring carcinogens.

The One Health implications extend beyond human food safety. Aflatoxins reduce animal productivity, impairing feed conversion and immune function in poultry and swine, thereby undermining food security. Moreover, mycotoxin contamination of crops reduces marketability, contributing to economic losses and waste in low- and middle-income countries. Because fungi respond sensitively to temperature and humidity, climate change is altering fungal ecology, shifting aflatoxin risk zones northward into regions previously unaffected (66). This climate-driven expansion confirms the necessity of integrated crop-animal-human monitoring systems that combine meteorological modeling, fungal genomics, and toxicovigilance.

Other mycotoxins, such as ochratoxin A, fumonisins, and zearalenone, illustrate similar cross-domain linkages. Fumonisins disrupt sphingolipid metabolism and cause leukoencephalomalacia in horses and have been associated with esophageal cancer in humans, while zearalenone acts as a potent estrogenic disruptor across livestock and humans (112, 113). The resulting health effects are not isolated events but manifestations of a shared biochemical vulnerability to fungal secondary metabolites across species.

Advancing One Health management of mycotoxins requires harmonized surveillance across the entire agroecosystem. The European Food Safety Authority (EFSA) and FAO/WHO Joint Expert Committee on Food Additives (JECFA) have emphasized the use of integrated sentinel monitoring, leveraging livestock as early detectors of feed contamination. Innovations in omics-based biosensors and predictive fungal ecology modeling now allow preemptive identification of high-risk regions before outbreaks occur. In this context, One Health toxicology becomes an enabler of precision prevention, linking plant pathology, veterinary health, and public nutrition into a single continuum of safety governance.

#### 5.2.4 Pesticide and endocrine-disruptor exposure

Pesticides and endocrine-disrupting chemicals (EDCs) exemplify how anthropogenic compounds designed for utility in one species can inadvertently interfere with endocrine and developmental pathways conserved across the biosphere. Originally developed to increase agricultural productivity and control vectors of disease, pesticides are now among the most ubiquitous chemical stressors, with residues detectable from Arctic ice cores to human breast milk. Evidence shows EDC exposure is associated with a broad range of adverse health outcomes, including metabolic diseases, cardiovascular disorders, and neurodevelopmental effects, reinforcing their relevance to One Health risk assessment (60, 157). The One Health perspective reframes them not simply as occupational or food-safety concerns but as agents of ecosystem-wide hormonal perturbation.

#### 5.2.5 Cross-species endocrine disruption

Endocrine disruptors act at extremely low doses by mimicking, antagonizing, or otherwise altering the synthesis, transport, and metabolism of natural hormones. Many, such as organochlorine pesticides (e.g., DDT, aldrin), organophosphates (e.g., chlorpyrifos), and triazine herbicides (e.g., atrazine), can interfere with endocrine signaling through receptor-mediated and non-receptor-mediated mechanisms in organisms as diverse as amphibians, birds, mammals, and fish (114, 115). Comprehensive scientific reports from the Endocrine Society and IPEN summarize global evidence that EDCs such as PFAS, bisphenols, and certain pesticides pose significant risks to reproductive, metabolic, neurological, and immune systems in wildlife and humans (116). Their interference with estrogenic, androgenic, and thyroidal signaling underlies a spectrum of effects from reproductive malformations and feminization in amphibians to neurobehavioral and metabolic disorders in humans.

Perhaps the most striking ecological sentinel of endocrine disruption is the observation of intersex fish populations downstream of wastewater-treatment effluents and agricultural catchments (117). Males of several freshwater species exhibit vitellogenin expression and ovarian tissue development, reflecting chronic exposure to mixtures of estrogenic compounds including synthetic contraceptive residues, alkylphenols, and agricultural chemicals. These effects presaged growing recognition that some EDCs can display non-monotonic dose–response relationships and critical developmental windows of vulnerability, a paradigm shift that challenges traditional toxicology models.

#### 5.2.6 Agricultural intensification and human health

From a human perspective, pesticide exposures span occupational, dietary, and ambient pathways. Agricultural workers are often the first and most severely affected, manifesting acute cholinergic crises from organophosphate or carbamate poisoning and chronic outcomes such as neurodegenerative and reproductive disorders (118). Dietary exposure through residues in fruits, vegetables, and grains is nearly universal, albeit at lower concentrations; however, cumulative risk assessment indicates that additive and synergistic interactions among multiple compounds can produce significant endocrine and neurodevelopmental effects even below regulatory thresholds.

Emerging epidemiological data link prenatal or early-life exposure to specific pesticide classes with attention-deficit/hyperactivity disorder, autism-spectrum behaviors, thyroid dysfunction, and reduced IQ (118–120). Such findings illustrate that endocrine and neurodevelopmental axes are shared across mammalian biology, reinforcing the One Health principle that protecting vulnerable human populations requires minimizing ecosystem contamination at its source.

#### 5.2.7 Ecological cascades and biodiversity loss

The ecological repercussions of pesticide overuse extend far beyond target species. Neonicotinoid insecticides, while effective against crop pests, impair navigation, immunity, and reproduction in pollinators such as honeybees and bumblebees (121). Their systemic persistence in nectar and pollen links agricultural chemical management directly to global declines in pollinator populations and subsequent threats to food security. Similarly, amphibians exposed to sub-lethal concentrations of herbicides such as atrazine exhibit demasculinization, immunosuppression, and increased susceptibility to parasitic infections (122). These cascading effects on non-target fauna demonstrate how endocrine disruption acts as an ecological force multiplier, eroding biodiversity and ecosystem resilience.

### 5.3 Air, water and soil pollution, climate change, and multi-stressor exposure

#### 5.3.1 Mixtures, climate change, and planetary feedbacks

Modern exposure scenarios rarely involve single compounds; rather, they consist of complex chemical mixtures interacting with nutritional, infectious, and climatic stressors. Climate change compounds pesticide risk by expanding pest ranges, increasing volatilization, and prolonging the persistence of certain compounds, thereby intensifying application frequency and environmental load (66). Warmer and wetter conditions also enhance microbial degradation of some chemicals into equally or more toxic metabolites and interconnect with climate change (59, 123). In aquatic ecosystems, higher temperatures accelerate uptake kinetics, further stressing thermally sensitive species (43). These feedbacks highlight that endocrine disruption must be considered within the broader planetary-health nexus linking agriculture, climate, and ecology.

#### 5.3.2 Air

Ambient and indoor air pollution represent some of the most ubiquitous exposure pathways for humans, animals, and plants alike, with particulate matter (PM₂.₅ and PM₁₀), ozone, nitrogen dioxide, and volatile organic compounds (VOCs) penetrating deep into respiratory systems across species (53). A 2024 systematic review confirmed that air pollution drives systemic inflammatory and oxidative stress pathways that are mechanistically linked to cardiovascular, respiratory, neurological, and metabolic outcomes in both human cohorts and animal models, illustrating the cross-species fidelity of air toxicity pathways (53). Wildfire smoke, intensifying under climate change, has emerged as a particularly acute multi-stressor exposure, releasing not only particulate matter but also polycyclic aromatic hydrocarbons (PAHs), dioxins, and heavy metals that persist in ashfall and enter soil and water systems far from the fire front (66). Recent research on the intersections of climate change and the chemical exposome highlights how elevated ground-level ozone and altered atmospheric transport patterns shift pollutant fate and widen geographical exposure windows, particularly affecting rural and resource-limited communities (59). The indoor environment, often overlooked in ecological risk assessment, is a critical intersection for One Health: pets, livestock, and humans share indoor air spaces where combined exposure to cleaning agents, pesticides, VOCs from building materials, and combustion products creates additive and synergistic toxicological burdens. Sentinel species such as birds (highly sensitive to aerial toxins) provide early indicators of ambient air quality degradation that can foreshadow human health effects, operationalizing the One Health surveillance principle in the atmospheric compartment.

#### 5.3.3 Soil

Soil functions as the primary environmental reservoir and long-term archive of chemical contamination, simultaneously affecting plant health, animal nutrition, groundwater quality, and human exposure through food, water, and dust inhalation (69). Heavy metals, including lead, cadmium, arsenic, chromium, and mercury, rank among the most widespread and persistent soil contaminants globally, disrupting soil microbial communities and nutrient cycling while bioaccumulating through food chains from crops to livestock to human consumers (69). A 2024 meta-analysis examining the impact of microplastic pollution on soil greenhouse gas emissions found significant increases of 60% in methane and 12% in carbon dioxide from microplastic-contaminated soils, revealing a previously underappreciated climate–soil–pollution feedback loop (124). An equally important and counterintuitive interaction was documented in 2025: carbon sequestration efforts in agricultural soils, such as those promoted under the Four Per Mille Initiative - may inadvertently increase the mobilization risk of metal(loid)s, as organic matter amendments alter soil pH and redox conditions in ways that enhance metal bioavailability (125). This finding illustrates the necessity of whole-system risk assessment in land-management decisions, where climate mitigation and chemical safety objectives can conflict. Bioremediation and soil amendment strategies are increasingly evaluated not only against crop yield or water quality endpoints but also against indicators of animal and human health co-benefit, reflecting the integrative logic of One Health toxicology.

#### 5.3.4 Water

Water bodies act as the ultimate integrator of terrestrial, agricultural, and industrial chemical loads, concentrating pharmaceuticals, heavy metals, pesticides, and microplastics into dynamic exposure matrices that span human, animal, and ecosystem health (167). Climate change amplifies water contamination risks through multiple pathways: heat waves and prolonged droughts alter the concentration and transformation of pharmaceuticals in surface waters, and the resulting sub-inhibitory antibiotic concentrations can drive selection for resistance, while altered precipitation patterns concentrate pollutants in shrinking water bodies and increase the frequency of combined sewer overflow events (135). The co-occurrence of heavy metals and antibiotics in aquatic environments creates a synergistic resistance hazard: sublethal metal concentrations upregulate bacterial efflux pump genes, simultaneously enhancing resistance to both metals and antibiotics, and bioavailable metal fractions are now recognized as better predictors of antibiotic resistance gene abundance in freshwater systems than total metal concentrations (127–129, 162). A 2025 systematic review (135) found that climate change and antimicrobial resistance (AMR) constitute interconnected threats requiring integrated One Health solutions, and One Health stewardship frameworks identify wastewater treatment plants as critical control points where clinical, agricultural, and environmental resistomes converge (130, 134). The endocrine-disrupting potential of waterborne chemical mixtures, including synthetic estrogens from contraceptive residues, alkylphenols from industrial surfactants, and PFAS from aqueous film-forming foams, further demonstrates the cross-species vulnerability of aquatic exposure, as evidenced by the widespread observation of intersex fish downstream of wastewater effluents (117). These convergent lines of evidence call for cumulative risk assessment frameworks that evaluate water quality not as an isolated environmental compartment but as the shared exposure medium connecting human clinical outcomes, livestock and aquaculture health, and ecosystem integrity.

### 5.4 Antimicrobial resistance and depression as transdisciplinary case studies for the One Exposome

#### 5.4.1 Antimicrobial resistance as a chemical–biological exposure system

AMR is a pressing One Health issue, with the misuse of antibiotics in livestock, aquaculture, and crop protection driving the emergence of resistant bacterial strains and transferable resistance genes. The World Health Organization Resistance Global Action Plan on AMR (168) and regional frameworks such as the U.S. National Action Plan (131) and the European One Health AMR strategy^26^ promote cross-sector data sharing, stewardship programs, and phased reductions in agricultural antibiotic use.

Recent global surveillance data from the WHO Global Antibiotic Resistance Surveillance Report 2025^27^ show that one in six common bacterial infections is now resistant to standard antibiotic treatments globally, with resistance rising across more than 40% of key pathogen-antibiotic combinations, revealing the accelerating threat of AMR. This trend affects urinary, gastrointestinal, bloodstream, and sexually transmitted infections and highlights the need for integrated global monitoring and One Health interventions (126).

Emerging solutions to address AMR include metagenomic surveillance, wastewater-based epidemiology, alternatives to antibiotics such as bacteriophage therapy, probiotics, and vaccination strategies, and environmental treatment technologies to degrade antimicrobial residues. The integration of micro- and macro-evolutionary perspectives into ecotoxicology can improve predictions and long-term assessment of ecosystem health, and the use of lake sediment archives can provide valuable insights into the impacts of aquatic pollution. The AMR crisis demonstrates that toxicology and microbiology are converging disciplines, with the selective pressure imposed by antibiotics constituting a chemical form of ecological toxicity that operates at the molecular level of microbial genetics but manifests as a global public health threat. Contemporary One Health frameworks emphasize that AMR spans clinical, agricultural, and environmental domains, requiring cross-cutting surveillance systems, stewardship across the entire antimicrobial lifecycle, and integration of environmental microbiomes into risk assessment and policy planning (130).

Antimicrobial resistance epitomizes the chemical–biological convergence that defines 21st-century One Health challenges. Unlike conventional toxicants, antimicrobials are bioactive agents intentionally designed to alter microbial physiology, but their misuse transforms them into ecological pollutants with evolutionary consequences. Excessive or prophylactic use of antibiotics in livestock, aquaculture, and crop protection creates selective pressures that foster resistant bacterial strains and transferable resistance genes (15, 132, 133, 167). These resistant organisms and genetic determinants disseminate through food, water, and soil, bridging the microbial ecosystems of farms, hospitals, and natural environments.

Within aquaculture systems, where antibiotics are frequently administered via feed or water, resistant bacteria colonize sediments and aquatic biota. These serve as reservoirs for mobile genetic elements such as plasmids and integrons that encode multi-drug resistance. River sediments receiving effluents from pharmaceutical manufacturing or wastewater treatment plants further act as genetic exchange hubs, sustaining complex microbial consortia capable of transferring resistance across species and habitats (134). Climate change interacts with AMR dynamics: warming temperatures, altered precipitation, and increasing extreme weather events may accelerate the spread of resistant microbes via water systems and food supplies, compounding AMR burdens especially in vulnerable regions. This climatic amplification of AMR highlights the importance of integrating environmental shifts into One Health strategies (135).

The human health implications are profound. Resistant pathogens arising in livestock can infect farm workers, spread via food supply chains, and re-enter clinical settings as zoonotic resistant strains of *Salmonella*, *Campylobacter*, or *Escherichia coli* (131). In parallel, the environmental release of sub-inhibitory antibiotic concentrations drives horizontal gene transfer and persistence of resistance even in the absence of direct clinical exposure. The resulting “*antibiotic resistome*” represents a planetary-scale biochemical experiment where anthropogenic molecules reshape microbial evolution.

Addressing AMR therefore demands integrated surveillance and stewardship across medical, veterinary, and environmental sectors, a foundational One Health principle. A recent 2024 definition of antimicrobial stewardship from a One Health perspective underscores the need to coordinate human, animal, and ecological stewardship efforts to minimize unnecessary antibiotic use, optimize therapy, and preserve antimicrobial efficacy across domains (136).

The World Health Organization Global Action Plan on AMR (168), complemented by regional frameworks such as the U.S. National Action Plan (131) and the European One Health AMR strategy, explicitly promote cross-sector data sharing, stewardship programs, and phased reductions in agricultural antibiotic use. Yet implementation remains uneven: antibiotics critical to human medicine continue to be deployed as growth promoters in some countries, and environmental monitoring of antimicrobial residues remains sporadic.

Emerging solutions illustrate the practical value of a One Health approach. These include:

- *Metagenomic surveillance* to map resistomes across environmental, animal, and human microbiomes;
- *Wastewater-based epidemiology* to detect and track resistance hotspots;
- *Alternatives to antibiotics*, such as bacteriophage therapy, probiotics, and vaccination strategies that reduce disease burden without chemical interventions; and
- *Environmental treatment technologies*, including advanced oxidation and constructed wetlands, to degrade antimicrobial residues before they enter waterways.

Ultimately, the AMR crisis demonstrates that toxicology and microbiology are converging disciplines. The selective pressure imposed by antibiotics constitutes a chemical form of ecological toxicity, i.e., one that operates at the molecular level of microbial genetics but manifests as a global public health threat. As with other One Health toxicology cases, success depends not only on technological innovation but on governance reform, aligning agricultural practice, environmental management, and clinical stewardship under a shared planetary ethic of chemical restraint.

#### 5.4.2 The Environome of depression

Depression provides a useful One Health Exposome case study because it is not determined by biology alone, but emerges from interacting biological, social, environmental, and digital exposures across the life course. Within the One Exposome framework, health is shaped by measurable environments and by the integration of these data into predictive and preventive systems. In this perspective, depression can be understood as a multideterminant outcome of the environome: a dynamic constellation of personal, social, natural, built, and digital exposures acting on interconnected complex systems (137, 158).

This interpretation aligns with the role of NAMs in One Health toxicology. NAMs provide a scientific infrastructure for linking molecular initiating events to organismal and ecological outcomes, supporting cross-species extrapolation and predictive prevention. Applied to mental health, this implies that depression should be studied not only through clinical or behavioral categories, but also through exposure patterns, mechanistic pathways, and environmental risk architectures.

A One Health toxicology framing is particularly relevant where environmental exposures and mental health outcomes converge through conserved mechanisms. Human-relevant systems such as iPSC-derived neural cultures, brain organoids, and microphysiological systems can help test whether determinants identified by digital dashboards converge on pathways relevant to depression, including neuroinflammation, endocrine disruption, stress-response biology, synaptic function, mitochondrial dysfunction, and neuronal network activity. This may be especially important for early-life exposures and neurodivergent populations, where vulnerability and symptom presentation can differ from the general adult literature.

This framing positions depression within a broader environmental health matrix. Pollutants may contribute to mental health burdens through oxidative stress, endocrine disruption, and neuroinflammation, while sentinel species may reveal early warnings of neurotoxic or endocrine-disrupting exposures before full human impacts are visible. If mental health is partly environmentally mediated, then upstream surveillance of pollutants, ecosystem disruption, and shared environmental matrices becomes part of mental health prevention rather than a separate policy domain.

### 5.5 Sentinel surveillance and early-warning systems

Sentinel surveillance is central to a One Exposome approach because non-human organisms can reveal environmental hazards before their full impact becomes evident in human populations. Wildlife, livestock, companion animals, and ecosystem indicators may manifest early signs of toxicity through conserved biological pathways, including oxidative stress, endocrine disruption, immunotoxicity, neurotoxicity, and reproductive impairment. Monitoring these signals can therefore transform ecological observation into a predictive public health tool.

Persistent contaminants rarely remain confined to their original context of use. PFAS, flame retardants, pesticides, heavy metals, pharmaceuticals, and microplastics can circulate through air, water, soil, food webs, and human-made environments, creating networked exposure pathways across species and ecosystems. Climate change can further modify these pathways by altering pollutant fate, transport, persistence, and biological uptake, thereby generating multi-stressor conditions relevant to One Health toxicology (59).

In this context, sentinel species and environmental matrices provide complementary forms of early warning. Wildlife morbidity, altered reproductive patterns, antimicrobial resistance signals in wastewater, contaminant accumulation in sediments, and biomarker changes in exposed organisms can indicate emerging risks before they are detected through conventional human health surveillance. When integrated with human biomonitoring, environmental monitoring, veterinary data, and exposomic analytics, these signals can support earlier intervention and more targeted prevention.

The value of sentinel surveillance depends on cross-sector integration. Human health, veterinary, environmental, food-safety, and chemical-risk systems often operate separately, creating blind spots in the detection of shared hazards. A One Exposome framework addresses this fragmentation by linking biological sentinels, environmental measurements, and population health data within interoperable surveillance systems. Such systems can support early detection, cumulative risk assessment, and prevention-oriented decision-making across human, animal, and ecosystem health.

### 5.6 From risk prediction to prevention and intervention

Traditional pesticide regulation, grounded in single-compound risk thresholds, struggles to capture such multi-layered, cross-species impacts. One Health toxicology advocates a cumulative-risk framework that integrates human biomonitoring, wildlife surveillance, and environmental residue mapping into a shared data infrastructure. Sentinel species, such as amphibians, fish, or pollinators, serve as early warning systems for human exposure, while AOP models translate mechanistic findings across *taxa*.

Interventions must also bridge policy silos. Coordinated efforts among agricultural ministries, public-health agencies, and environmental regulators can promote Integrated Pest and Pollinator Management (IPPM) strategies (138), reduction of high-risk active ingredients, and substitution with biological or mechanical controls; recent initiatives such as the European Commission’s One Health fact-finding visits to Member States (170) illustrate growing efforts to bridge these silos, while also documenting that multisectoral ambition at national level is often diluted at regional and local levels. At the research frontier, microphysiological systems (MPS) replicating endocrine tissues and neurodevelopmental pathways are providing new mechanistic insights into low-dose mixture effects (71, 78), complementing field observations and supporting mechanistic risk assessment within the One Health framework.

In sum, pesticide and endocrine-disruptor exposures illustrate how chemical convenience can evolve into biological ubiquity. Their control demands not only toxicological innovation but also social transformation, shifting agriculture toward sustainability, strengthening global regulation of persistent pollutants (110), and embedding environmental ethics into economic policy (139). As such, they complete the One Health toxicology typology: from heavy-metal legacies and mycotoxins to antimicrobial and pesticide resistance, these cases collectively define a continuum of shared chemical vulnerability that unites species, ecosystems, and societies.

The convergence of MPS, AI, and exposomics defines the next frontier of One Health toxicology. Future priorities include:

1. *Mechanistic integration:* Mapping full AOP networks across species to unify human and ecological health endpoints.
2. *Digital twins and simulation:* Building AI-driven models that replicate organismal and population-level responses to exposure (106).
3. *Regulatory co-creation:* Establishing adaptive validation frameworks where regulators, scientists, and technology developers collaborate in real time (68).
4. *Data equity and access:* Ensuring global interoperability and open science in NAM-generated data to prevent technological monopolization (55).
5. *Planetary relevance:* Extending NAM principles to climate- and biodiversity-linked chemical risks, aligning with the Sustainable Development Goals (59).

As NAMs and AI mature, toxicology’s scope will extend from assessing isolated compounds to modeling the complex chemical ecosystems that shape the exposome, the cumulative lifetime environmental exposure of humans and other species. This integrative vision embodies the very ethos of One Health: science serving as the connective tissue between biological, societal, and planetary systems.

The maturation of a One Health Intelligence System will redefine prevention itself. Integration of eco-exposomes, FAIR data, digital twins, and inclusive governance will allow early detection of environmental perturbations, targeted remediation, and policy foresight. AI-enabled analytics can identify “*tipping points*” where cumulative chemical stress intersects with climate or socio-economic vulnerability, triggering proactive interventions long before thresholds of harm are reached.

In this vision, the HEP is not merely a data initiative but a planetary health observatory, a self-learning system aligning molecular biology, ecology, and ethics in continuous feedback. Where One Health articulates the moral imperative of interconnected life, the exposome and NAMs deliver the measurable, mechanistic, and computational infrastructure to sustain it.

## 6 Ethical, regulatory, and implementation challenges

### 6.1 Bias, transparency, reproducibility, and model validation

The synergy of NAMs, MPS, and AI redefines validation: not by concordance with animal data but by *mechanistic plausibility* and *human relevance*. “*E-validation*” (92) and “*fit-for-purpose qualification*” (140) form the backbone of HEP quality assurance. Together with GCCP 2.0 (80, 141), GIVIMP (88) and GIVReSt (89, 142, 143), they provide reproducibility, reliability, relevance, transparency, and FAIR data stewardship, preconditions for global exposome analytics.

- *Validation Ethics and Evidence-to-Decision:* Validation in exposomics is not only methodological but moral. Deciding what counts as credible evidence determines whose experiences count as real. *Validation ethics* (68) reframes reliability as a virtue of transparency, probabilistic reasoning, and human relevance rather than animal concordance.
- *Evidence-to-Decision (EtD) framework*: To translate evidence into policy, the EtD provides a transparent pathway weighing benefit, harm, equity, feasibility, and societal values. Applied to exposome data, it transforms ethical reasoning into public-health action, i.e., the final step from information to justice (68).

Limitations and failure modes of AI in the One Exposome (159). The advantages of AI must be weighed against well-documented limitations that become especially consequential when models inform regulatory or public-health decisions. Algorithmic bias can arise where training data over-represent high-income settings, particular ancestries, or well-studied chemicals, yielding models that systematically under-serve the populations and exposures most in need. Limited explainability of complex models constrains mechanistic interpretation and erodes the trust required for regulatory acceptance; explainable and human-supervised AI is therefore a prerequisite rather than an optional refinement. Data quality and provenance remain rate-limiting, because heterogeneous, sparse, and non-standardized exposure data propagate uncertainty that is easily masked by confident model outputs. Transparency (of data, code, model versioning, and performance across subgroups) is necessary for reproducibility and independent scrutiny. Finally, accountability must be assigned: when an AI-supported inference contributes to a regulatory or clinical decision, responsibility for error, redress, and monitoring cannot be delegated to the algorithm. We regard these as first-order design constraints for the One Exposome, to be met through documented validation, uncertainty quantification, bias auditing, and clear human oversight, rather than as caveats to be noted and set aside.

### 6.2 Privacy, data sovereignty, and community consent

The One Exposome framework raises ethical challenges because it integrates biospecimens, geolocation data, environmental measurements, wearable-sensor streams, omics profiles, and AI-derived predictions. These data are often re-identifiable and socially revealing, requiring governance models that go beyond technical anonymization (70).

Key ethical safeguards include:

- *Privacy and data sovereignty:* individuals and communities should retain meaningful control over access, reuse, and interpretation of their exposure and health-related data.
- *Dynamic consent and participant engagement:* consent should evolve from a one-time procedure into an ongoing process, allowing participants to update preferences, withdraw, re-consent, and receive understandable feedback.
- *AI accountability and exposome justice:* predictive models should be transparent, explainable, validated, and assessed for bias, so that AI supports prevention rather than reproducing inequities (79, 92, 144).
- *Sensors, commerce, and surveillance:* wearables, environmental sensors, app-based risk scoring, and commercial exposure services can support prevention, but also create risks of surveillance, commodification, and unequal access.

For these reasons, One Exposome data infrastructures should be governed as a public good, grounded in transparency, proportionality, community participation, equitable benefit-sharing, and prevention-oriented action.

### 6.3 Environmental justice and unequal exposure burdens

*Equity and Environmental Justice:* Ethical legitimacy depends on justice by design. Historically marginalized communities often bear the highest exposure burdens yet the least research agency. The *One Exposome* approach demands Community Advisory Boards (CABs) with real decision-making power, co-created research agendas, and equitable benefit-sharing. Ethical success is measured not by publications but by empowerment: whether exposomic evidence helps communities secure remediation and policy reform.

#### 6.3.1 Global equity and capacity building

The promise of One Exposome is global, but participation remains uneven. Low- and middle-income countries (LMICs) face disproportionate environmental burdens yet often lack the infrastructure to measure, model, or mitigate them (139). Remarkably, the Global Exposome Forum (see Footnote 9) has started to address this by initiating regional chapters in Africa, South America, India, and China, and its partnerships with UNESCO and the International Network for Government Science Advice (INGSA) further strengthen the global approach. Without deliberate investment, the exposome revolution risks deepening-not reducing-global health inequities.

Equitable participation means enabling LMICs to become data producers, not merely data providers or consumers. This requires:

- Regional Exposome Hubs equipped with modular MPS laboratories, open analytical platforms, and affordable omics instrumentation;
- Cloud-based toolkits and mobile applications for real-time environmental sensing and biomonitoring;
- Training pipelines that integrate toxicology, data science, and regulatory policy; and
- Reciprocity clauses in international data-sharing agreements guaranteeing local co-ownership, authorship, and technology transfer.

True global equity also requires attention to the social determinants of exposure, urbanization, industrial activity, and socio-economic factors, which can amplify health risks even within well-characterized environmental compartments.

#### 6.3.2 Capacity building and equity in low- and middle-income countries

For the One Health Intelligence System to serve as a global public good, capacity building in low- and middle-income countries (LMICs) is essential. Historically, environmental health data have been geographically skewed toward high-income regions, leaving large parts of the Global South underrepresented in exposure and toxicology datasets (139). Equitable participation requires infrastructure investments in laboratory capability, data governance, and training in bioinformatics, AI, and exposomics.

Partnerships through the NIEHS NEXUS Network, EIRENE, and the Global Exposome Forum can facilitate open-source toolkits, modular MPS platforms, and cloud-based analytics accessible to resource-limited settings. Embedding reciprocity clauses in data-sharing agreements, ensuring local benefit, co-authorship, and technology transfer, will institutionalize fairness and sustainability. In this way, global exposome science becomes not extractive but co-creative, aligning with the ethics of shared stewardship outlined in Charting Exposomethics (70).

### 6.4 Regulatory acceptance of AI and NAM-based evidence

Implementing NAMs within a One Health framework also transforms regulatory and ethical landscapes. The paradigm shifts from reactive testing toward proactive prevention, from vertebrate use to *mechanistic evidence*, and from compliance-driven validation to continuous learning (99).

Ethically, NAMs fulfill the 3Rs principle (replacement, reduction, refinement) (145) while enabling a *fourth R*: relevance. The societal momentum toward phasing out animal testing, reinforced by legislation such as the U.S. FDA Modernization Act 2.0 and European chemical-safety reforms, aligns with the maturation of NAMs as potentially more human-relevant alternatives for defined contexts of use (145).

For global policy, establishing One Health NAMs consortia, linking human toxicology, veterinary pharmacology, and ecological monitoring, could standardize mechanistic biomarkers, promote data transparency, and embed AI ethics within safety decision-making frameworks. These structures would transform NAMs from tools into governance mechanisms for sustainable chemical management.

### 6.5 Regulatory readiness and policy translation

Perhaps the most formidable frontier is regulatory readiness—the incorporation of exposome-based evidence into chemical-safety, food-safety, and public-health decision making (164). Traditional risk assessment frameworks are ill-suited to dynamic, multi-source data streams and probabilistic modeling outputs. Regulators must therefore transition from deterministic thresholds toward evidence-weighted, adaptive decision systems (68).

The Evidence-to-Decision (EtD) framework provides one path forward (50), integrating benefit–risk balance, feasibility, and equity into transparent deliberations. Regulatory agencies could pilot living risk dossiers, where AOP-anchored NAM data, digital-twin simulations, and real-time biomonitoring continually update exposure guidance (92).

Inter-agency coordination is essential. The Quadripartite One Health Joint Plan of Action (146) identifies chemical pollution and antimicrobial resistance as cross-cutting priorities, yet implementation remains fragmented. Establishing One Health Regulatory Networks-joint review committees that include environmental, agricultural, and health authorities-could accelerate policy harmonization and data exchange. Regulatory frameworks must also explicitly link mechanistic knowledge of chemical exposures (e.g., antibiotics, pesticides, endocrine disruptors) to preventive interventions, ensuring that toxicological insights are actionable across humans, animals, and ecosystems.

Finally, regulatory readiness must extend to ethical and social governance. The validation of exposome-driven NAMs must be accompanied by validation ethics (68), emphasizing transparency, uncertainty communication, and stakeholder participation. Public trust will hinge on clear articulation of how AI-derived predictions translate into protective action, avoiding both technocratic opacity and political inertia.

#### 6.5.1 Regulatory transformation and anticipatory governance

The shift from static to adaptive science demands a parallel transformation in regulation. Future governance must move beyond compliance with legacy test requirements toward anticipatory, evidence-based decision frameworks informed by real-time exposome intelligence (68, 139).

The EtD model already applied in exposome ethics (68) provides a scaffold for this evolution, embedding transparency, equity, and feasibility into policy translation. Continuous integration of AOP-anchored NAM data and digital-twin simulations can enable living risk assessments, i.e., dynamic dossiers that evolve with new evidence rather than awaiting crises for revision. Regulatory sandboxes, co-developed between agencies, academia, and industry, can pilot these adaptive frameworks, building public trust through openness and explainability.

Ultimately, this anticipatory governance completes the feedback loop: data inform prediction; prediction informs prevention; prevention reduces exposure; and reduced exposure feeds back as improved planetary health.

#### 6.5.2 Policy and governance implications

Coming back to our introductory example: Following recognition of diclofenac’s ecological effects, India, Nepal, and Pakistan banned its veterinary use and adopted meloxicam (3). Yet diclofenac remains one of the most frequently detected pharmaceuticals in European surface waters (147). Harmonized global governance linking chemical, veterinary, and environmental regulation is required.

The Quadripartite (WHO–FAO–WOAH–UNEP) *One Health Joint Plan of Action* (2022–2026)^28^ provides a policy scaffold, prioritizing antimicrobial resistance, food safety, and chemical pollution as trans-sectoral threats.

The European Commission’s Joint Research Centre (JRC) has experience in data science integrating One Health approaches,^29^ and is internationally recognised for its work in the context of emerging health threats and public health policy. These research and policy activities explicitly focus on applying One Health principles, which address the interconnectedness of human, animal, and environmental health, by developing standardized frameworks, supporting EU disease surveillance strategies, and advancing non-animal testing in toxicology. This is in line with the EU Group of Chief Scientific Advisors report.^30^

### 6.6 Governance of AI-supported One Health decision-making

#### 6.6.1 Ethical and governance resonances

The HEP amplifies One Health’s moral core: interdependence implies shared responsibility. Ethical foresight must therefore accompany technological progress. Three interlinked domains have been proposed:

- *Bioengineering ethics:* dynamic consent for human-derived cells, governance of organoid constructs, and limits to sentience (70).
- *Exposome ethics*: privacy, data sovereignty, environmental justice, and participatory governance (70).
- *Regulatory risk ethics:* decision-making under uncertainty using probabilistic, transparent validation rather than animal concordance (139).

Embedding these principles into HEP operations ensures that the technological unification of One Health is matched by ethical coherence.

#### 6.6.2 Ethics as infrastructure for a shared planetary health

The *One Exposome* vision, to map and mitigate the totality of exposures shaping human and planetary health, cannot succeed without a parallel ethical architecture. Just as *One Health* integrates the biological interdependence of species and ecosystems, *One Exposome* must integrate the moral interdependence of data, power, and justice. The exposome’s scope (linking biospecimens, wearable sensors, geolocation traces, and socio-environmental data) blurs traditional boundaries between public and private, individual and collective, research and surveillance.

As outlined in *Charting Exposomethics: A Roadmap for the Ethical Foundations of the Human Exposome Project* (70), exposome research will generate unprecedented, fine-grained, multimodal datasets. These hold immense promise for prevention, but also profound risks of privacy loss, inequity, and exploitation. Ethics in the exposome era therefore shifts from protection *after the fact* to governance *by design*.

This chapter articulates a framework for “Exposome Ethics by Design,” embedding justice, transparency, and accountability throughout the research lifecycle from data generation and AI modeling to policy translation. It situates exposome ethics as the moral counterpart to scientific infrastructure: as necessary for trust and legitimacy as laboratories or biobanks are for data.

#### 6.6.3 From bioethics to Exposomethics

Traditional bioethics evolved around individual autonomy and clinical consent. In contrast, *exposomethics* operates within a collective and ecological moral field. Whereas genomics demanded rules for individual genetic privacy, exposomics demands principles for *environmental and communal data sovereignty*.

The Human Genome Project’s ELSI (Ethical, Legal, and Social Implications) framework established vital precedents but remains anchored in individualism. Exposome ethics must instead encompass the *collective dimension*, recognizing that exposures are shared phenomena rooted in social structures and geography. The unit of moral concern is not only the person, but the community, the ecosystem, and the data networks that bind them.

- *Governance and Oversight:* Institutional Review Boards (IRBs) are ill-equipped for multi-omic, sensor-driven exposome research. Specialized Exposome Ethics Review Panels and an International Exposome Ethics Consortium (IEEC) are proposed to develop harmonized guidelines for dynamic consent, AI accountability, and global data sharing. Governance must include *adaptive oversight*, periodic ethical audits, and participatory review structures. This is governance not as bureaucracy, but as a living social contract.
- *Actionability and Responsible Communication:* Exposome findings are often probabilistic and context-dependent. Ethics requires distinguishing four types of actionability (personal, clinical, community, and policy-level) and tailoring communication accordingly. Reporting an exposure without feasible means of remediation risks stigmatization or despair. Conversely, withholding findings that could mobilize collective action is paternalistic. Ethical communication should therefore aim at empowerment without burden, guided by transparent Evidence-to-Decision (EtD) frameworks, notably a 100 + member working group of the Evidence-based Toxicology Collaboration (EBTC).^31^
- *Program Goals and Planetary Responsibility:* Finally, the ultimate ethical question is not *can* we collect these data, but *to what end*? Research objectives must align with public and planetary health needs, avoiding extractive or commercial exploitation. Ethics thus becomes directional: defining where the Human Exposome Project should go, not merely how it should behave.

*Building an Ethical Infrastructure for One Exposome:* Realizing *One Exposome* requires ethics as infrastructure is planned, funded, and institutionalized. We propose three operational pillars:

- *Participatory Governance:* Community Advisory Boards with decision authority over data use, interpretation, and dissemination.
- *Embedded Ethics Research:* Parallel ethical inquiry embedded within exposome projects, monitoring consent, trust, and justice outcomes in real time.
- *Dedicated Oversight:* National and international Exposome Ethics Consortia coordinating standards, training IRBs, and maintaining open repositories of ethical case studies.

#### 6.6.4 Toward an ethics of anticipation

The ethics of One Exposome moves beyond protectionism to anticipatory governance. It is not merely about avoiding harm, but about enabling fairness, participation, and empowerment in a data-intensive world.

Ethical foresight must accompany technological foresight. As the Human Exposome Project advances, its legitimacy will depend not only on scientific innovation but on *moral imagination*, the ability to design systems that respect autonomy while nurturing collective agency.

Just as ELSI became inseparable from genomics, *Exposomethics* must become inseparable from the Human Exposome Project. Embedding ethics as a constitutive infrastructure, through dynamic consent, community governance, and AI accountability, ensures that One Exposome fulfills its promise:

*to measure what matters, protect what connects us, and translate knowledge into planetary justice.*

One Exposome extends the 3Rs of animal welfare into a broader ethical framework for environmental justice and participatory governance. The *Exposome Ethics* framework (70) emphasizes privacy, data sovereignty, inclusivity, and benefit-sharing across populations and species. Validation ethics (68) replaces rigid animal concordance with human-relevant, probabilistic trust. In this sense, One Exposome is not only a technological construct but a social contract, a commitment to transparency, equity, and stewardship of shared planetary health.

## 7 Research gaps and future directions

The accelerating convergence of NAMs, MPS, AI, and exposomics (20) is creating the foundation for what can be envisioned as a One Health Intelligence System (79), a dynamic, self-updating network capable of detecting, explaining, and ultimately preventing chemical- and environment-driven disease across species, ecosystems, and populations.

Unlike classical toxicology, which relies on static datasets and retrospective hazard attribution, a One Health Intelligence System integrates real-time sensing, mechanistic modeling, and predictive simulation into a living evidence architecture. Its goal is to transform One Health from a guiding philosophy into an operational, data-driven infrastructure for planetary prevention.

### 7.1 Building interoperable One Exposome data ecosystems

Achieving global equity and reproducibility requires open, FAIR^14^ (Findable, Accessible, Interoperable, Reusable) data infrastructures. The emerging Global Exposome Commons envisioned by the Exposome Moonshot Forum^17^ is designed to democratize access to multi-omics, environmental, and clinical exposure data (71).

This commons would federate distributed databases, such as clinical biobanks, ecological observatories, and citizen-science platforms, under privacy-preserving governance (79, 160). Federated learning and secure cloud architectures can allow algorithmic training on sensitive datasets without moving raw data, maintaining sovereignty while enabling discovery (144). Open science is not only a technical principle but an ethical one (50, 70): it ensures that the benefits of exposome intelligence accrue globally rather than concentrating within data-rich regions.

Despite rapid conceptual and technological advances, realizing the full potential of the One Exposome paradigm will require overcoming several structural and epistemic barriers. These challenges are not merely technical-they are cultural, organizational, and ethical. They reflect the need to transform how science produces, governs, and translates knowledge across species, disciplines, and societies. The One Exposome paradigm spans multiple environmental compartments, including air, water, soil, and food systems, capturing the continuum of chemical, biological, and physical stressors that link ecosystem and human health (20, 79).

Practical barriers to implementation. Realizing the One Exposome will require confronting a set of concrete, and partly unsolved, implementation challenges. Data interoperability across environmental, human, animal, and AI-generated sources is still limited by incompatible formats, ontologies, and consent regimes, and is the most common single point of failure for integrative programs. Infrastructure requirements (federated storage, computation, secure record linkage, and long-term stewardship) imply sustained institutional funding that project-based mechanisms rarely provide. Validation of AI and NAM models for regulatory use is still maturing, and fit-for-purpose validation frameworks, reference datasets, and performance standards are needed before their evidence can be relied upon. Regulatory acceptance depends on that validation but also on inter-agency coordination and legal mandates that currently lag behind the science. Implementation costs (for instrumentation, curation, computation, and workforce) are substantial and unevenly distributed, and feasibility in low-resource settings cannot be assumed: without deliberate capacity building, regional hubs, and reciprocal data-sharing, the framework risks widening rather than narrowing global exposure inequities. We present these not as reasons for delay but as the practical agenda whose resolution will determine whether the One Exposome is adopted; several are developed further in the equity, governance, and validation sections below.

### 7.2 Combining mechanistic toxicology with AI-driven prediction

The transition from descriptive to predictive toxicology requires the systematic integration of mechanistic insight across molecular, organismal, and ecological levels of organization. Traditional hazard evaluation relies largely on phenomenological endpoints, e.g., mortality, organ pathology, or reproductive failure, derived from high-dose animal tests. These endpoints, while historically useful, rarely reveal why a substance causes harm or how those effects propagate across species and ecosystems.

The One Health paradigm reframes toxicology as an information science of shared pathways of injury, linking cellular perturbations to population-level effects and embedding those connections within environmental and social contexts. To operationalize this shift, three interlocking mechanistic frameworks have emerged: AOPs (148), Sentinel Surveillance (73) and Cumulative Assessment (104), and Systems Toxicology (77) integrated with Exposomics (20, 21). Together, they form the conceptual and computational backbone of a modern, One Health–aligned toxicology.

#### 7.2.1 Adverse outcome pathways

The AOP framework formalizes mechanistic knowledge from a molecular initiating event (MIE), such as receptor binding, enzyme inhibition, or oxidative stress, through successive key events (KEs) leading to an adverse outcome (AO) measurable at the organism or population level (149). This modular structure provides a transparent, evidence-based map of causal relationships across scales of biological complexity.

AOPs facilitate mechanistic reasoning in both regulatory and research contexts by linking perturbations across species via conserved biological pathways. For instance, renal prostaglandin inhibition leading to urate precipitation, proposed as central to diclofenac-induced vulture mortality (1, 4), is mechanistically similar to renal toxicity observed in mammals, illustrating how avian-specific AOPs – had they been developed – could have supported earlier recognition of this ecological catastrophe.

The strength of the AOP concept lies in its scalability and transferability. Individual pathways can be combined into AOP networks that represent entire domains of toxicity, such as endocrine disruption, neurotoxicity, or immunotoxicity, across *taxa* (148, 150). These networks, curated in collaborative databases such as the AOP-Wiki^32^ and OECD Effectopedia (151), serve as public repositories for regulatory science and NAM development.

The integration of AOPs with omics-driven discovery and computational modeling now allows quantitative prediction of toxic outcomes. Quantitative AOPs (qAOPs) incorporate dose–response data, toxicokinetics, and feedback regulation to simulate thresholds and probabilities of adverse effects (150). When embedded in NAMs, AOPs provide the causal skeleton that translates molecular data from *in vitro* or *in silico* systems into regulatory endpoints without reliance on animal testing (152, 153).

In the One Health context, AOPs enable cross-species extrapolation and environmental risk forecasting, allowing regulators to connect mechanistic evidence from sentinel species or laboratory systems to ecosystem-level protection goals. They are thus both scientific frameworks and policy instruments, aligning mechanistic toxicology with sustainable, predictive regulation.

### 7.3 Moving from correlation to causality

Collectively, AOPs, sentinel surveillance, and systems toxicology form an integrative mechanistic triad for One Health toxicology. AOPs provide the conceptual grammar of causality (100); sentinel surveillance supplies the empirical data of exposure and effect; and systems toxicology translates both into quantitative, predictive models.

Embedding these tools within an exposomic framework transforms toxicology from a static discipline into a dynamic intelligence system, capable not only of explaining how toxicants harm but of anticipating where and when harm will occur. In doing so, One Health toxicology unites mechanistic science, ecological monitoring, and digital innovation into a single transdisciplinary enterprise aimed at protecting all life forms connected through shared chemical and biological environments.

### 7.4 Developing public-health-ready digital twins

Digital-twin technology (computational avatars calibrated with MPS, omics, and sensor data) is an emerging approach that, if adequately validated, could contribute to the predictive engine of the One Health Intelligence System (79, 106). At the individual level, digital twins could simulate organ-specific or systemic responses to complex exposure mixtures, enabling precision prevention and adaptive risk management. At the population level, cohorts of digital twins can model environmental interventions, policy scenarios, or climate-exposure interactions, guiding anticipatory public-health strategies (71).

These models are envisioned to be self-learning and, in principle, continuously refined through feedback from real-world biomonitoring and sentinel data. Coupled with explainable AI (xAI) methods (107, 108, 144), digital-twin infrastructures could provide transparent, probabilistic forecasts that regulators and communities alike can interpret and act upon.

### 7.5 Building the next generation of One Exposome scientists

All these frontiers converge on one decisive need: people. The future of One Exposome depends on cultivating scientists fluent in multiple languages-biology, computation, policy, and ethics. Graduate programs, professional societies, and funders must foster transdisciplinary curricula that bridge toxicology, environmental science, AI, and data governance (68, 71). Fellowships and online academies co-organized by CAAT (expanding our COURSERA classes^33^^,^^34^ or similar, (154)), IHEN, and regional networks could create an agile, globally connected workforce. Training should explicitly integrate monitoring and mechanistic prevention strategies across air, water, soil, and food compartments, linking computational modeling with real-world interventions.

Addressing data heterogeneity, standardization, equity, and regulatory readiness will require coordinated investment in infrastructure, training, and ethics as robust as the technologies themselves. The reward is profound: a science of prediction and prevention that operates across species and societies. By transforming these challenges into design principles, the One Exposome community can move from vision to implementation-building a planetary health intelligence system that is as inclusive and dynamic as the environment it seeks to protect.

Testable propositions and measurable outcomes. Although the One Exposome is a conceptual framework rather than a single hypothesis, its value can and should be evaluated empirically, and we therefore propose several testable propositions and measurable outcomes by which future studies could judge whether it improves environmental-health surveillance and decision-making. First, integrative prediction: One Exposome models that combine environmental, human, and sentinel-species data should outperform single-domain models in predicting adverse outcomes, measurable as gains in sensitivity, specificity, and calibration against held-out cohorts. Second, early warning: cross-species sentinel surveillance should shorten the lead time between an emerging exposure and its recognition, measurable as the interval between a sentinel signal (for example, in wildlife or companion animals) and a confirmed human effect. Third, mechanistic concordance: NAM- and AOP-anchored predictions should agree with observed human and ecological outcomes at rates exceeding conventional read-across, testable against curated benchmark exposures. Fourth, decision impact: incorporating One Exposome evidence into Evidence-to-Decision workflows (50) should measurably change prioritization, monitoring, or regulatory action, evaluable through prospective case studies with pre-registered decision criteria. Fifth, equity: deployment should reduce, not amplify, disparities in exposure characterization across regions and populations, measurable through coverage and performance metrics stratified by setting (70). Framing these propositions makes the One Exposome falsifiable in practice and defines a concrete research agenda for its validation.

## 8 Conclusion

The conceptual progression from One Health to the One Exposome reflects a broader transformation in environmental health science: from fragmented assessment to systems integration, from animal-based toxicology to human-relevant biology, and from descriptive data collection to actionable knowledge. Together, these developments support a common epistemology of interconnection, in which human, animal, and ecosystem health are understood as interdependent and shaped by shared environmental conditions.

One Health provides the ethical and ecological rationale: health is shared across species, communities, and ecosystems. The exposome provides the measurement framework: health-relevant exposures are cumulative, multidimensional, and life-course dependent. NAMs, including MPS, provide the mechanistic and human-relevant experimental infrastructure needed to interpret exposure–response relationships. AI and digital health technologies provide the computational layer required to integrate heterogeneous data, detect patterns, support prediction, and inform prevention-oriented decisions.

The One Exposome paradigm emerges from the convergence of these elements. It is not simply an extension of exposome science, nor only an operational expression of One Health. Rather, it is proposed as an integrating conceptual framework that aspires to more predictive, preventive, participatory, and equitable environmental health. It seeks to make environmental exposures more visible, mechanistically intelligible, and actionable across biological, ecological, and societal scales. At present these remain proposed capabilities: the degree to which the framework actually improves prediction, prevention, and governance is a hypothesis to be tested rather than an established outcome.

The InDepDash depression dashboard (see *The Environome of Depression*)^35^ exemplifies how this perspective can be operationalized, structuring heterogeneous, environome-level evidence into a policy-oriented tool that links environmental, social, and biological determinants of disease. Depression is therefore a compelling out-of-the-box One Health example of why the One Exposome is more than an environmental monitoring agenda. It demonstrates the need to move beyond downstream treatment and toward upstream prevention, integrating exposure surveillance, mechanistic understanding, population vulnerability, and evidence-informed governance. A One Exposome approach can support the identification of neglected determinants, the recognition of vulnerable populations, and the development of interventions that are not only clinically informed, but also environmentally and socially grounded.

The eco-exposome perspective further expands this logic by recognizing that exposure is shared across environmental compartments, species, and communities. Climate-related disruption, pollution, ecosystem degradation, and changes in the built environment should therefore be considered not as externalities, but as health determinants. This perspective supports climate-informed public health planning, justice-centred data governance, and interoperable infrastructures capable of linking environmental, biological, and social signals.

In this perspective, the HEP can be viewed as a systems-science counterpart to the Human Genome Project. Whereas the genome helped reveal the biological code that contributes to individual uniqueness, the exposome addresses the shared and dynamic environmental conditions that shape health across populations and ecosystems.

The road forward requires sustained international cooperation, harmonized validation frameworks, transparent and explainable computational methods, and a strong commitment to open, interoperable, and ethically governed data infrastructures. These requirements are scientific as well as institutional: the One Exposome will only be effective if its tools are robust, its evidence base is inclusive, and its governance models are capable of addressing structural inequities and vulnerable populations.

Through this unified lens, toxicology can move beyond its historical role as the science of harm and become a science of foresight: an intelligence architecture for planetary resilience. One Health defines the ethical imperative; the exposome defines the measurable environment; NAMs and MPS provide the experimental and mechanistic means; AI provides the analytical capacity to integrate knowledge at scale.

The One Exposome therefore completes a conceptual triad: One Health explains why integrated health protection is necessary; NAMs and MPS show how human-relevant evidence can be generated; and the exposome defines what must be measured, interpreted, and acted upon. Their convergence signals a paradigm capable of translating environmental data into planetary-scale health intelligence, supporting a future in which risks are not only documented after harm occurs, but anticipated, understood, and mitigated before they become crises.

## Data Availability

The original contributions presented in the study are included in the article/Supplementary material, further inquiries can be directed to the corresponding author.

## Footnotes

1 The authors are most grateful for the article by Tim Ehlers in Katapult, February (156), “*Versehentlicher Geiergenozid kostete Hunderttausende Menschen das Leben*”, our translation “Accidental Vulture Genocide Costs Hundreds of Thousands of Human Lives”, which brought this premier example of One Health to our attention.

2 EXPOSIM: Environmental stressors as causal determinants for immune-mediated diseases, mapping and prioritizing evidence for knowledge-based policy-making https://exposim.eu

3
https://www.european-health-data-space.com

4
https://exposomemoonshot.org/declarationsigning/

5
https://www.nexus-exposomics.org

6
https://www.humanexposome.eu

7
https://eirene.eu

8
https://humanexposome.net

9
https://globalexposomeforum.org

10
https://impss.org

11
https://mpsworldsummit.org

12
https://www.oecd.org/en/publications/guidance-document-on-good-in-vitro-method-practices-givimp_9789264304796-en.html

13
https://commonfund.nih.gov/complementarie

14
https://www.go-fair.org/fair-principles/

15
https://aopwiki.org

16
https://impss.org

17
https://exposomemoonshot.org

18
https://www.oecd.org/en/topics/sub-issues/testing-of-chemicals/adverse-outcome-pathways.html

19
https://safecast.org

20
https://www.inaturalist.org

21
https://health.ec.europa.eu/ehealth-digital-health-and-care/european-health-data-space-regulation-ehds_en

22
https://hl7.org/fhir/

23 https://ohdsi.github.io/CommonDataModel/ · OHDSI: https://www.ohdsi.org/data-standardization/

24
https://smart.who.int/ddcc/

25
https://obofoundry.org

26
https://health.ec.europa.eu/system/files/2020-01/amr_2017_action-plan_0.pdf

27
https://www.who.int/publications/i/item/9789240116337

28
https://openknowledge.fao.org/items/fddae6a2-e7ef-4a2a-ad54-2463dbbb0b32

29
https://publications.jrc.ec.europa.eu/repository/handle/JRC138888

30
https://scientificadvice.eu/advice/one-health-governance-in-the-european-union/

31
https://www.ebtox.org

32
https://aopwiki.org

33
https://www.coursera.org/learn/toxicology-21

34
https://www.coursera.org/learn/evidence-based-toxicology

35
https://publications.jrc.ec.europa.eu/repository/handle/JRC139918
