# Supplementary material for: Towards the One Exposome: harnessing artificial intelligence and digital health technologies across One Health toxicology, new approach methodologies, and environmental public health
Source: Front Public Health. 2026 Oct 6;14:1921273. doi: 10.3389/fpubh.2026.1921273 (PMC13680456; doi:10.3389/fpubh.2026.1921273)
Supplement: Supplementary file 1 [file Table_1.docx]

Supplementary Material

Supplementary Material 1.
Complete key concepts for **Towards the One Exposome: Harnessing Artificial Intelligence and Digital Health Technologies across One Health Toxicology, New Approach Methodologies, and Environmental Public Health**

**Supplementary Table S1. Glossary of Key Concepts**

| **Term** | **Definition** |
| --- | --- |
| **One Exposome** | An integrated framework that extends the human exposome into a **One Health** context by linking **human, animal, and environmental exposures** across the life course. It combines **exposomics, AI, NAMs, and digital health technologies** to support prediction, prevention, and cross-species risk assessment across ecological, biological, and social systems . |
| **One Health Toxicology** | A **systems-based toxicology** approach that studies chemical and pollutant effects across **species and environmental compartments**, rather than in humans alone. It emphasises **shared mechanisms, conserved pathways, and linked exposure routes** among humans, animals, and ecosystems to support more preventive and ecologically realistic risk assessment . |
| **New Approach Methodologies (NAMs)** | **Non-animal, mechanistic methods** such as *in vitro* systems, organ-on-chip platforms, computational models, and omics-based tools that can assess exposure and toxicity with greater biological relevance. In the One Exposome framework, NAMs generate data for **predictive toxicology, regulatory decision-making, and reduced reliance on animal testing** . |
| **Microphysiological Systems (MPS)** | Advanced **human-relevant experimental systems**, including organ-on-chip and related platforms, that reproduce organ or tissue functions to generate multidimensional toxicological data. These systems are presented as a major source of mechanistic evidence within NAM-based and AI-supported One Health toxicology . |
| **Adverse Outcome Pathway (AOP)** | A **mechanistic framework** linking a **molecular initiating event** to a sequence of **key biological events** ending in an adverse outcome. In the paper’s framing, AOP networks help connect mechanistic findings across species and support the regulatory use of NAMs in One Health toxicology . |
| **Human Exposome** | The totality of **environmental exposures across the life course**, linked to behaviour and internal physiology. In the paper, it is treated as the conceptual foundation that is expanded into the broader **One Exposome** framework . |
| **Eco-Exposome** | The extension of exposome thinking to **shared environmental matrices** such as **air, water, soil, food webs, and ecosystems**, including exposure pathways that affect both humans and non-human organisms. It supports integrated monitoring of environmental contamination and cross-species health risks . |
| **Sentinel Surveillance** | A One Exposome strategy in which **non-human organisms, environmental matrices, and ecological indicators** are monitored as **early-warning systems** for environmental hazards before full human impacts are apparent. Examples include wildlife morbidity, reproductive changes, antimicrobial resistance signals in wastewater, and contaminant accumulation in sediments . |
| **Environmental Sentinel** | A **species, organism group, or environmental indicator** that signals emerging environmental risk through early biological or ecological change. The paper highlights wildlife, livestock, companion animals, wastewater signals, sediments, and exposed organisms as useful sentinels for shared toxicological threats . |
| **Cross-Species Extrapolation** | The use of **conserved biological pathways and mechanistic evidence** to infer toxicological effects across taxa. Within One Health toxicology, this supports the transfer of insight between humans, animals, and ecosystems when exposure pathways and biological responses are linked . |
| **Exposomic Analytics** | Analytical approaches that integrate diverse exposure-related data, including biomonitoring, environmental, omics, and digital data, to identify exposure patterns and support prediction. In the paper, AI-driven exposomic analytics are described as helping operationalise One Health at the systems level . |
| **AI-Enabled Toxicology** | The application of **artificial intelligence** to multidimensional data from NAMs, omics, imaging, biosensors, and exposure monitoring in order to derive **mechanistic understanding, predictive models, and decision support**. It is framed as the integrative layer needed to handle the scale and complexity of One Exposome data . |
| **Digital Twin** | An **AI-driven simulation model** of an organismal or population-level response to exposure, used to explore how chemicals and stressors may affect biological systems over time. The paper identifies digital twins and simulation as a future opportunity for One Health toxicology . |
| **Cumulative Risk Assessment** | A broader form of risk evaluation that considers **complex chemical ecosystems**, multiple exposures, and interacting stressors rather than isolated compounds. The One Exposome vision aims to move toxicology towards modelling these cumulative and interconnected exposure realities . |
| **Digital Health Intelligence** | The transformation of heterogeneous exposome-related data into **actionable knowledge for prediction, prevention, and environmental public health** through interoperable digital infrastructures. It depends on connecting health, environmental, veterinary, and biodiversity data while addressing semantic, ethical, and governance interoperability . |
| **Digital Health Integration Platforms** | Software and data infrastructures that connect **heterogeneous One Exposome data streams** such as health records, biomonitoring, environmental monitoring, and other digital sources into usable public health intelligence. They are presented as essential for operationalising integrated exposome surveillance . |
| **European Health Data Space (EHDS)** | A European regulatory and technical framework for **secure access, exchange, and secondary use of electronic health data**. In the paper, it is cited as a model for broader interoperable infrastructures that could link health, biomonitoring, environmental, veterinary, wildlife, and sentinel-species data within a One Exposome approach . |
| **Federated Data Architecture** | A distributed data infrastructure in which analysis can occur across multiple institutions without centralising all underlying data. The paper highlights federated architectures as especially relevant for One Exposome research because they help preserve **privacy, institutional control, and data sovereignty** . |
| **Citizen Science** | Public participation in data collection and environmental observation through tools such as reporting platforms and sensor-based initiatives. Within exposome surveillance, citizen science can extend monitoring beyond conventional institutions, provided that quality assurance and ethical safeguards are in place . |
| **Participatory Epidemiology** | Community-based collection and reporting of environmental and health-related observations through portals or applications that contribute to public health intelligence. The paper presents this as part of distributed exposome surveillance, subject to validation, bias assessment, and appropriate governance . |
| **Dynamic Consent** | An adaptive ethical approach to consent for data use, particularly relevant where longitudinal, sensitive, and evolving exposome data are collected. The paper identifies **dynamic consent** as part of the ethical requirements for human-derived materials and exposome-related governance . |
| **Data Sovereignty** | The principle that individuals, communities, or institutions retain meaningful control over how their data are accessed, used, and governed. It is highlighted as a core ethical issue in exposome research alongside privacy, environmental justice, and participatory governance . |
| **Exposome Ethics** | The ethical framework needed for exposome research, especially given its use of **biospecimens, wearable sensors, geolocation traces, and socio-environmental data**. The paper identifies central concerns including **privacy, data sovereignty, environmental justice, and participatory governance** . |
| **Regulatory Risk Ethics** | An ethical approach to decision-making under uncertainty that favours **probabilistic and transparent validation** rather than simple concordance with animal tests. It is presented as part of governance for AI-supported and NAM-based One Health decision-making . |
| **3Rs and Relevance** | The ethical principle that NAMs support **replacement, reduction, and refinement** of animal use, while also enabling a fourth dimension: **relevance**, meaning better scientific fit to human and ecological biology. The paper presents this as part of the ethical and regulatory shift accompanying One Health NAMs . |
| **Mechanistic Integration** | The effort to connect mechanistic evidence across endpoints and species, including the mapping of **full AOP networks**, in order to unify human and ecological health assessment. The paper identifies this as a major future priority for One Health toxicology . |
| **Regulatory Co-Creation** | An adaptive validation approach in which **regulators, scientists, and technology developers collaborate in real time** to shape methods and evidence standards. It is proposed as a future model for implementing AI- and NAM-based toxicology . |
| **Data Equity and Access** | The principle that One Exposome data systems should promote **global interoperability and open science** while avoiding exclusion and technological monopolisation. The paper identifies this as a future priority in the governance of NAM-generated and AI-ready data . |
| **Planetary Relevance** | The extension of toxicological assessment beyond isolated human endpoints to include **climate-linked and biodiversity-linked chemical risks** affecting shared ecosystems. The paper presents this as part of aligning One Health toxicology with sustainable development goals and ecological reality . |
| **Quadripartite One Health Joint Plan of Action** | An international policy framework developed by **FAO, UNEP, WHO, and WOAH** to advance collaboration on human, animal, plant, and environmental health. It is cited in the paper’s references as a governance touchstone relevant to broader One Health alignment . |
